# Surface manipulation for prevention of migratory viscous crude oil fouling in superhydrophilic membranes

**DOI:** 10.1038/s41467-023-38419-3

**Published:** 2023-05-09

**Authors:** Yuanyuan Zhao, Xiaobin Yang, Zhongjun Cheng, Cher Hon Lau, Jun Ma, Lu Shao

**Affiliations:** 1 MIIT Key Laboratory of Critical Materials Technology for New Energy Conversion and Storage, State Key Laboratory of Urban Water Resource and Environment, School of Chemistry and Chemical Engineering, Harbin Institute of Technology, Harbin, PR China; 2 School of Engineering, The University of Edinburgh, The King’s Buildings, Edinburgh, UK; 3 School of Environments, Harbin Institute of Technology, Harbin, PR China

**Keywords:** Pollution remediation, Synthesis and processing, Chemical engineering

## Abstract

Here, we present a proactive fouling prevention mechanism that endows superhydrophilic membranes with antifouling capability against migratory viscous crude oil fouling. By simulating the hierarchical architecture/chemical composition of a *dahlia* leaf, a membrane surface is decorated with wrinkled-pattern microparticles, exhibiting a unique proactive fouling prevention mechanism based on a synergistic hydration layer/steric hindrance. The density functional theory and physicochemical characterizations demonstrate that the main chains of the microparticles are bent towards Fe^3+^ through coordination interactions to create nanoscale wrinkled patterns on smooth microparticle surfaces. Nanoscale wrinkled patterns reduce the surface roughness and increase the contact area between the membrane surface and water molecules, expanding the steric hindrance between the oil molecules and membrane surface. Molecular dynamic simulations reveal that the water-molecule densities and strengths of the hydrogen bonds are higher near the resultant membrane surface. With this concept, we can successfully inhibit the initial adhesion, migration, and deposition of oil, regardless of the viscosity, on the membrane surface and achieve migratory viscous crude oil antifouling. This research on the PFP mechanism opens pathways to realize superwettable materials for diverse applications in fields related to the environment, energy, health, and beyond.

## Abstract (web summary)

Minimizing membrane fouling is critical for their continuous and efficient operation. Here, authors demonstrate a wrinkled-pattern microparticles-decorated membrane surface can increase contact area with water molecules, expand steric hindrance, and inhibit adhesion and deposition of oil, achieving efficient migratory viscous crude oil antifouling.

## Introduction

Oily sewage poses a significant threat to vulnerable ecosystems and can degrade the global water-food-energy nexus and water-soil-waste connection^1–3^. Developing superhydrophilic and superoleophobic underwater materials is an efficient strategy to realize oil-in-water emulsion separation^2,4–7^ and has been applied in industrial oily sewage treatment^8–10^. The basic separation principle mainly relies on superhydrophilicity, where water can smoothly pass through the pore structures and gaps inside the material to form a hydration layer on the material surface and prevent the passage of oil. Then, the capillary force generated by the micro and small pores and the high specific surface area can produce emulsion-demulsification, which is conducive to emulsion separation^11–14^. However, fouling is the main factor restricting the application of superhydrophilic materials. The introduction of hydrophilic functional groups and increasing the surface roughness can help improve the water affinity of material surfaces, thereby improving the fouling resistance^15^.

Many studies have focused on the preparation of superhydrophilic materials by coating hydrophilic particles on the surfaces of materials and increasing the surface roughness to reduce oil fouling and extend service life^16–18^. The current state-of-the-art superhydrophilic materials possess the conventional “passive resistance to fouling” (PRF) mechanism. The key limitation of the PRF mechanism is that it prevents only the initial nonmigratory adsorption on the surface. It cannot effectively block the adsorption, deposition, diffusion, migration, and coalescence of migratory oil droplets^11,12^. In addition, the increased surface roughness inevitably increases the risk of hidden oil droplets and fouling in the gullies on the surfaces of the materials^19,20^. Thus, scientists have developed and applied wrinkling models on the surfaces of hydrophilic particles coated on superhydrophilic materials to further improve the oil fouling resistance and separation performance^21^. Based on the Wenzel mode, wrinkling on the particles of superhydrophilic materials facilitates the spread and infiltration of water droplets, which can further enhance the superhydrophilicity. Water can rapidly penetrate the materials to form a continuous and stable water layer in the air–water–solid three-phase interface and reduce the contact area between the oil molecules and the material surface, effectively delaying oil fouling and improving the separation performance^22^.

The mechanism of wrinkling formation has been intensively investigated following the initial reports on artificial microwrinkled patterns on double-layer membranes^23–25^. In nature, the ubiquitous wrinkling structures on planar and curved biological surfaces such as white blood cells, gecko toes, hydrangea L. seeds, and rose petals display unusual wettability^26^. Some studies have been inspired by these wrinkling structures and have used them to prepare superhydrophilic materials^27–29^. For example, Li et al. utilized wrinkled graphene monoliths to reversibly switch between superhydrophobicity and superhydrophilicity to achieve fast oil/water separation^21^. Although wrinkle formation on the surfaces of particles has been studied, the mechanism by which wrinkling on the particle surface improves the fouling resistance and separation performance is still unclear. Another classic natural surface with wrinkled structure is the *dahlia* leaf enriched with caffeoyl ester groups, comprising papillae as the essential microsubstrate with the cuticle on the microstructure folded into wrinkled patterns. This leads to superhydrophilicity with a water contact angle of 0°, where water is quickly absorbed and spread out^30–32^.

**Fig. 1:**
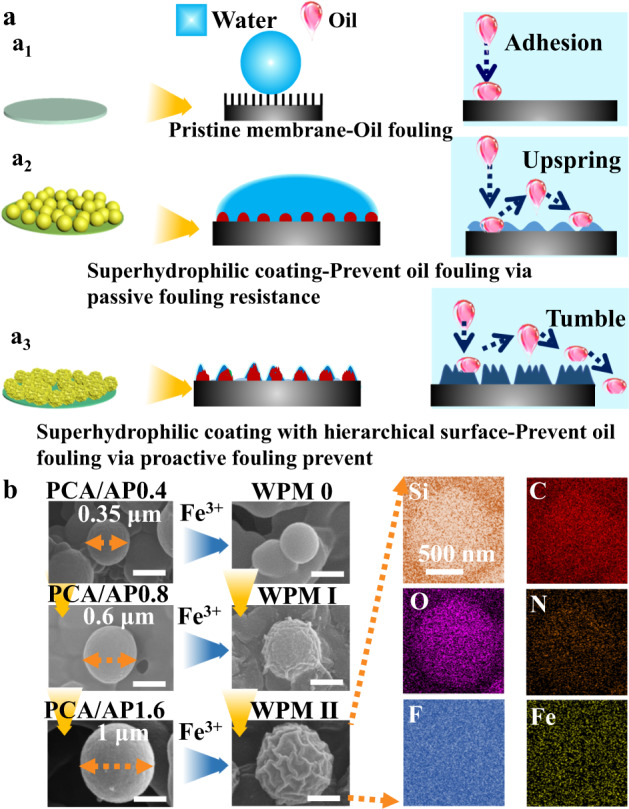
Schematic illustration of the mechanism to prevent oil fouling of membranes. **a** Schematic of the proposed mechanism for preventing oil fouling of membranes: **a**_**1**_ Pristine membrane adheres to oil droplets; **a**_**2**_ Superhydrophilic membrane prevents oil droplets following the “passive resistance to fouling (PRF)” mechanism; **a**_**3**_ Superhydrophilic membranes with wrinkled patterns repel oil droplets, following the “proactive fouling prevention” (PFP) mechanism. **b** SEM images showing the wrinkling of the surface morphologies of the PCA/AP smooth hydrophilic microparticles following Fe^3+^ complexation (scale bar: 500 nm) and EDS mapping of a WPM II microparticle showing the fine dispersion of Si, C, F, O, N, and Fe throughout the microparticle. PCA/APX: Hydrophilic microparticles are formed by the polymerization of caffeic acid (CA) and (3-aminopropyl) triethoxysilane (APTES), WPM X: The wrinkled patterns of microparticles are depicted on the smooth PCA/AP surface.

In this work, inspired by the superhydrophilicity of *dahlia* leaves, we hypothesize that well-tuned wrinkles on microsubstrates can be utilized to significantly reduce the force between the surface and the adhered viscous crude oil. Consequently, crude oil can be easily removed with the application of a shear force, establishing a crude oil antifouling mechanism. As proof of this concept, we develop smooth hydrophilic microparticles with wrinkled patterns and coat them on porous microfiltration membranes (MF), establishing atypical superhydrophilicity underpinned by a “proactive fouling prevention” (PFP) mechanism that inhibits the adsorption, migration, and aggregation of viscous crude oil droplets (see Fig. 1a). In detail, based on the traditional PRF mechanism, smooth hydrophilic microparticles are formed by the polymerization of caffeic acid (CA) and different amounts (0.4, 0.8, and 1.6 g) of (3-aminopropyl) triethoxysilane (APTES) and are named PCA/AP0.4, PCA/AP0.8, and PCA/AP1.6, respectively. These are coated on primary superhydrophilic membranes (PCA/APX MFs) (see Fig. 1b). Then, the wrinkled patterns are depicted on the smooth PCA/AP surface. Such microparticles are denoted as wrinkle-patterned microparticles (WPM X), and the modified membranes comprising such microparticles with different degrees of surface wrinkling are denoted as WPM X MFs. Fe^3+^ modification does not alter the morphology and shape of PCA/AP0.4 with a diameter of 0.35 µm. We denote such microparticles as WPM 0. Meanwhile, Fe^3+^ complexation transforms the smooth surfaces of PCA/AP0.8 with a diameter of 0.6 µm into rough-edged, wrinkled structures with ridges and craters (WPM I). Fe^3+^ complexation of PCA/AP1.6 with a diameter of 1.0 µm leads to labyrinth-like surface wrinkling (WPM II). The underwater oil contact angle (UOCA) of our WPM II MF exceeds 165° and is key to achieving a low underwater oil adhesive force (<0.5 µN). Thus, the mechanism of the formation of wrinkled patterns of WPM II MF to improve the crude oil fouling resistance and separation performance can be summarized as follows: First, reduce the surface roughness appropriately to expand the steric hindrance interactions between the membrane surface and oil molecules. Second, the contact area between the membrane surface and water molecules is increased to strengthen the water-molecule densities and the strengths of the hydrogen bonds (HBs) near the membrane surface. Such a tightly bonded hydration layer and steric hindrance bonds protect the membrane from crude oil fouling. In addition, the membrane enables efficient separation of crude oil/water emulsions with flux reaching 7247 L m^−2^ h^−1^ bar and rejection rates of 99.6%. This strategy has potential applications in dealing with oil spills and oil/water separation.

## Results

### Mechanism of surface wrinkling of smooth microparticles

As a low-cost micropatterning method, surface wrinkling has great application potential in intelligent wetting interfaces, artificial organs, biochemical protection, and stimulus-response devices. Therefore, inspired by the wrinkling model of *dahlia* leaves, a superhydrophilic membrane was fabricated by the step-coating method, as shown in Supplementary Table 1. First, 2 mg/ml CA was dissolved in a typical Tris-HCl solution (pH=8.5) in a petri dish containing MF for 1 h. Different amounts of APTES were poured into the CA solution for 12 h. Smooth hydrophilic PCA/AP was formed by the polymerization of CA and APTES (see Supplementary Fig. 1). Next, PCA/AP MFs were submerged into 100 ml FeCl_3_·6H_2_O solution for 1 h to form wrinkled patterns on the microparticles with defined sizes. The results of the size distributions of the microparticles (see Fig. 1b and Supplementary Fig. 2), pore-size distributions (see Supplementary Figs. 3–5), and morphologies of the corresponding modified membranes (see Supplementary Figs. 6–9) reveal that the size of the smooth PCA/AP microparticles increases from 0.35 to 0.6 and to 1.0 µm, respectively, as the APTES concentration increases. The pore size distribution of the corresponding membrane decreases gradually from 0.7 to 0.6 to 0.45 μm. The pore size and porosity of each WPM MF exhibit a weak upward trend compared to the corresponding PCA/AP MF, which is mainly due to the deformation of the microparticle surface after Fe^3+^ modification. Fe^3+^ complexation tailors the wrinkling of the smooth PCA/AP surface as a function of the microparticle radius. The wrinkled patterns generated on the surface of the smooth PCA/AP microparticles and the wrinkling model deepened with increasing microparticle size.

The wrinkling of surfaces is caused by the instability arising from the external stimulation of the spherical film/substrate systems, and the surface yield deformation of the system is spontaneously generated to regain the mechanical equilibrium and release the internal excessive stress (Σ)^32^. Theoretically, the wrinkling of curved substrates can be quantitatively characterized by the Swift-Hohenberg theory^32–34^. When the radius changes, the partial derivative of *σ*_*f*_ (the compressive hoop stress) with respect to the microparticle radius (*R*) can be obtained by:

1 $$\frac{\partial {\sigma }_{f}}{\partial {{{{{\rm{R}}}}}}}=\frac{E{E}_{s}({\alpha }_{s}-\alpha )\varDelta {{{{{\rm{T18}}}}}}({R}^{4}{{{{{\rm{h}}}}}}+2{R}^{3}{h}^{2}+{R}^{2}{h}^{3})[{E}_{s}{{{{{\rm{v}}}}}}+{{{{{\rm{E}}}}}}(1-2{v}_{s})]}{{[3{E}_{s}{R}^{3}(1-{{{{{\rm{v}}}}}})+{E}_{s}(1+{{{{{\rm{v}}}}}})(3{R}^{2}{{{{{\rm{h}}}}}}+{{{{{\rm{3R}}}}}}{h}^{2}+{h}^{3})+{{{{{\rm{2E}}}}}}(1-2{v}_{s})h(3{R}^{2}+{{{{{\rm{3Rh}}}}}}+{h}^{2})]}^{2}}$$

where *h* represents the film thickness, *E* represents the elastic modulus, Δ*T* represents the actual temperature change during deformation, *α* represents the coefficient of thermal expansion, *ν* represents Poisson’s ratio, and the subscripts *s* and *f* represent the substrate and film, respectively. Poisson’s ratio (*ν*), as the elastic constant of material transverse deformation, is generally between −1 and 0.5. As water is incompressible, the maximum value of Poisson’s ratio is 0.5. All microparticles developed in this work are compressible; therefore,

2 $$\nu \, < \,0.5$$

In addition, the chelation of Fe^3+^ ions with oxygen or nitrogen atoms is a typical exothermic reaction, which yields

3 $$\varDelta T \, > \,0$$

The formation of coordination bonds increases the ambient temperature, and the coefficients of thermal expansion will increase according to the spin state of the Fe^3+^ ions;^35,36^ thus,

4 $${\alpha }_{s}-\alpha \, > \,0$$

Substituting inequalities (2), (3), and (4) into Eq. (1), we deduce that

5 $$\frac{\partial {\sigma }_{f}}{\partial {{{{{\rm{R}}}}}}} \, > \,0$$

**Fig. 2:**
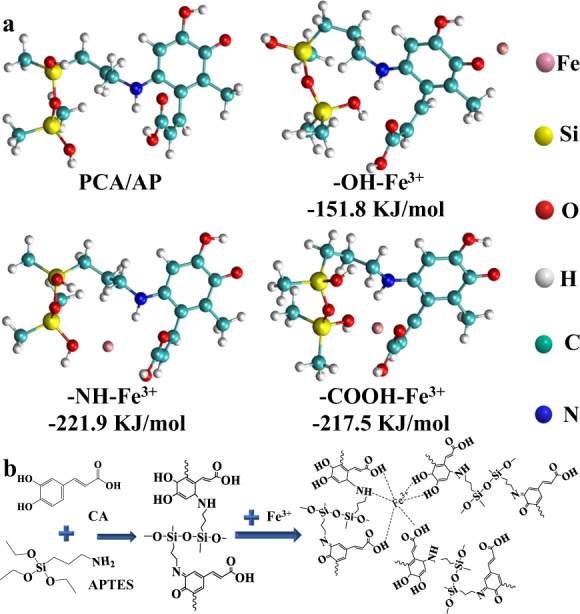
DFT calculations and proposed reaction mechanism of PFP. **a** DFT calculations of coordination between Fe^3+^ and PCA/AP. **b** Possible mechanism of ternary system (PCA, APTES, and Fe^3+^) coating on the membrane.

The result indicates that *σ*_*f*_ increases with respect to *R*, while Eqs. 6–8 (see Methods) show that *σ*_*c*_ (the critical wrinkled stress) decreases with respect to *R*. Hence, we conclude that Σ increases with a larger microparticle radius *R*. Wrinkles form spontaneously as the compressive stress in the spherical film/substrate systems surpasses *σ*_c_. In this work, this instability was caused by Fe^3+^ complexation on the microparticle surfaces. Here, we observe that the radius of 0.175 µm is insufficient to make *σ*_f_ greater than *σ*_c_ after Fe^3+^ complexation; hence, Σ <0. As a result, 0.35 µm hybrid microparticles do not undergo any surface transformation or deformation. As the average diameter of the microparticles increases by 71% to 0.6 µm, craters are formed on the surfaces of WPM I because microparticles with larger radii reduce the energy required to form such patterns. The amount of energy needed to stabilize these patterns is also lower^35,36^. As the radius of the microparticles increases to 0.5 µm, the geometric constraint becomes strong enough that the radius/excess stress of the microparticles increases^24^. This gathers the craters, huddling them into grooves that decrease the stretching energy, finally forming labyrinth-like wrinkled patterns. These hierarchical structures are similar to those found on *dahlia* leaves, which are closely related to hydrophilicity and antifouling performance. To elucidate the formation mechanism of wrinkled patterns on WPM II (see Supplementary Fig. 9) induced by Fe^3+^ complexation, a series of density functional theory (DFT) simulations were performed to evaluate the interactions between Fe^3+^ and PCA/AP (see Fig. 2a and Supplementary Table 2). Three types of coordination bonds are formed on the surfaces of WPM II upon introduction of Fe^3+^ ions. The binding energies of these bonds are −151.8 (-OH/Fe^3+^), −221.9 (-NH/Fe^3+^), and −217.5 kJ mol^−1^ (-COOH/Fe^3+^). The combined effect of these bonds causes the polymer chains of PCA/AP to bend towards Fe^3+^, causing conformational changes in the WPM II surface. Undoubtedly, without the coordination bonds, surface deformation of smooth microparticles does not occur even when their radii increase from 0.175 to 0.5 µm. Thus, surface wrinkling of microparticles occurs only when Fe^3+^-induced strong coordination bonds are coupled with optimal microparticle diameters. X-ray photoelectron spectroscopy reveals that the overall peak of the Fe 2p spectrum consists of two peaks centered at 726.5 and 712.5 eV. These peaks can be assigned to -N-Fe and -O-Fe, respectively, confirming the coordination between Fe^3+^ and the hydroxyl/amino groups^37^, which is validated through Fourier transform infrared spectroscopy (see Supplementary Figs. 10–12, Supplementary Tables 3, 4). The chemical compositions of the various membranes prove that Michael addition and Schiff base reactions lead to microparticle formation (see Fig. 2b)^38^. The enhancement of the hydrophilicity of the membrane surface has an important effect on creating resistance towards oils. Thus, further characterization of the effect of the wrinkled patterns of the microparticles on the membrane properties is necessary.

### Antiviscous crude oil fouling performance

**Fig. 3:**
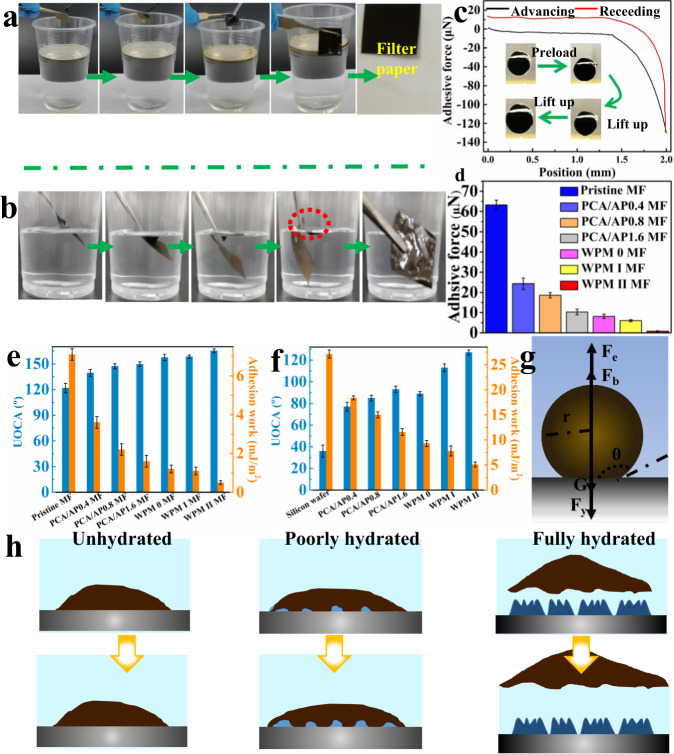
Crude-oil antifouling of membranes. Crude oil antifouling experiments of different membranes are demonstrated through a series of **a** dilute and **b** viscous crude oil experiments. **c** Real-time recorded force-distance curves during dynamic oil-adhesion measurements on WPM II MF. **d** Underwater crude oil adhesive forces of different membranes under a preloaded force of 50 μN. **e**, **f** Underwater crude oil angles (UOCA) and work of adhesion for different membranes and nascents. Error bars represent the standard deviations obtained from three membranes. **g** Force analysis of oil on the membrane surface under water. **h** Speculated mechanisms of crude oil adhesion or detachment from membrane surfaces. The blue color represents the water environment.

To evaluate the surface wettability, the water contact angle and UOCA of each membrane surface were measured^39–41^. Compared to the pristine MF, PCA/AP MFs exhibit a gradual increase in hydrophilicity (see Supplementary Fig. 13a). The superhydrophilicity of each WPM MF is greatly improved; in particular, water droplets penetrate the WPM II MF much faster than the PCA/AP1.6 MF (see Supplementary Fig. 13b). The UOCA of the pristine MF is 120°, whereas PCA/AP MFs exhibit higher underwater oleophobicity (see Supplementary Fig. 14). Furthermore, WPM MFs exhibit UOCAs greater than 150°, indicating superoleophobic surfaces. The UOCA of WPM II MF reaches 165 ° with good underwater oil repulsion (see Supplementary Figs. 15, 16). The results of the wettability stability of each membrane show that WPM MFs exhibit stronger and more stable superhydrophilicity than the original and PCA/AP MFs (see Supplementary Figs. 17–19). Superhydrophilicity and underwater superoleophobicity are closely related to anti-oil-fouling performance. Supplementary Fig. 20 illustrates that both PCA/AP1.6 and WPM II MF present defenses against low-viscosity oil, unlike the original MF. However, actual oily sewage often contains abundant crude oil; thus, the crude oil antifouling ability of the different membranes was investigated (see Supplementary Table 5). The dilute crude oil adheres to the original and PCA/AP1.6 MF, causing severe fouling. The contamination of WPM 0 and WPM I MF is partially relieved by water washing, while some crude oil still remains on the surface of the membrane. In contrast, WPM II MF does not retain any oil residue after a simple rinse (see Fig. 3a, Supplementary Fig. 22 and Supplementary Movie 1). Furthermore, after slight mechanical aviation via shaking, the viscous crude oil proactively lifts off from the WPM II MF surfaces into the water, achieving self-cleaning, unlike the other membranes where the crude oil adheres absolutely to the surface (see Fig. 3b, Supplementary Fig. 23 and Supplementary Movie 2). In a dynamic oil-adhesion experiment, no adhesion or deformation of the oil droplet was observed after it was pushed on or lifted up from the WPM II MF into water (see Supplementary Fig. 21). We tested the adhesive force of each membrane underwater to quantify their antiviscous crude oil fouling. When the viscous crude oil droplet was forced to fully contact the WPM II MF surface until the compressed oil droplet deformed on the surface, followed by lifting it up, no oil residue was observed on the WPM II MF surface (see Fig. 3c). The adhesive force during lift off was only 0.5 μN, showing the crude oil antiadhesion property (see Fig. 3d). To exclude the effect of wrinkled patterns of WPM II MF on the wettability and oil-droplet adhesion, the UOCA and work of adhesion were measured for a silicon wafer coated using the same proposed strategy as shown in Fig. 1a_3_ (see Fig. 3e, f). A larger UOCA demonstrates a lower work of adhesion, indicating a lower adhesion force. With the increase in APTES on the membrane and silicon wafer, the work of adhesion decreases gradually. The work of adhesion of WPM MFs is lower than that of PCA/AP MFs, indicating that the use of wrinkled patterns is conducive to weakening the adhesive force of oil droplets on the membrane surface. The work of adhesion of WPM II MF is the lowest, only 0.5 mg/m^2^ (93% less than that of the original MF). The work of adhesion on the silicon wafer is slightly higher, and the work of adhesion of WPM II MF is 5.1 mg/m^2^ (79% less than that of the original silicon wafer). This illustrates that while the inherent structure of the membrane has a certain influence on the adhesive force of oil droplets, the unique wrinkled patterns play a major role in reducing the adhesion force. Thus, WPM II MF can proactively prevent the initial adhesion of viscous crude oil and effectively prevent the migration, diffusion, and enrichment of oil fouling, which reveals an anti-oil-fouling strategy different from PRF mechanism, named “proactive fouling prevention” (PFP). PFP is mandatory for preventing fouling from viscous crude oil. To further investigate the PFP mechanism, we performed a free-falling experiment of oil droplets in water (see Supplementary Figs. 24, 25 and Supplementary Movie 3). The force analysis of a membrane surface underwater when oil droplets undergo free-falling in water and touch the membrane surface is presented in Fig. 3g. We can conclude that when the mass of the oil droplets and initial height is fixed, the adhesive force is related to the rebound height alone. When the rebound height is large, the adhesive force is small, illustrating the strength of the hydration layer. Therefore, we propose a PFP mechanism for WPM II MF (see Fig. 3h). Before being fouled by crude oil, the membranes are prewetted with water; hence, the ability to bond between water molecules and the membrane surface is the most important factor affecting the crude oil antifouling property. The crude oil adheres to the pristine MF after being washed with water, and the membrane surface exhibits serious fouling. Thus, an unhydrated layer is formed, and contact between crude oil and the membrane surface cannot be prevented. After immersion in water, there is no space for water molecules to be inserted between the surface and the adhered crude oil. After prewetting, owing to the smooth hydrophilic microparticles, the PCA/AP1.6, WPM 0, and WPM I MF surfaces bond weakly with water molecules, forming a poor hydration layer. Once crude oil touches the membrane surface, it immediately sticks. Then, the poor hydration layer is destroyed and is unable to form effective steric hindrance interactions. When immersed in water, water molecules cannot be inserted between the oil and the membrane surface, resulting in no relief from crude oil fouling. In contrast, the crude oil detaches easily from the surface in the case of WPM II MF, owing to the contact area between the water molecules and the membrane surface increasing greatly because of the nanoscale wrinkled pattern depicted on WPM II (see Supplemental Fig. 26). The hydration layer is tightly bound to inhibit the direct contact between the membrane surface and crude oil, forming strong steric hindrance interactions and a full hydration layer. Then, crude oil can be isolated from the membrane surface, and it floats on the water owing to its buoyancy, achieving crude oil antifouling performance.

### Proactive fouling prevention (PFP) mechanism

**Fig. 4:**
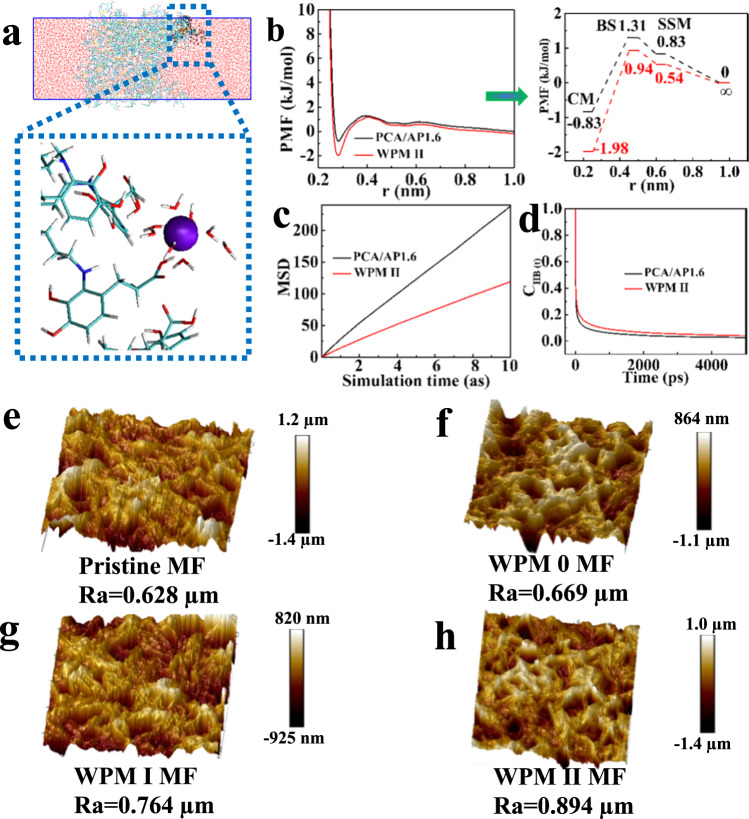
Proactive fouling prevention mechanism and separation performance of membranes. **a** Structure of the WPM II MF/water molecule with enhanced hydrogen bonds formed between the membranes and hydration layer. (The club-shaped structure represents PCA/AP, and the purple ball represents Fe^3+^). **b** Water absorption energy between PCA/AP/WPM II and water. PMF: Potential of mean force. **c**, **d** Diffusion coefficients and residence times of the hydration layer of PCA/AP and WPM II. MSD: Mean Square Displacement. HB: Hydrogen bonds. **e**–**h** AFM images of membranes.

The PFP mechanism is investigated based on two aspects. On the one hand, coordinate bonds play an important role in improving the viscous crude oil antifouling performance. To clarify the PFP mechanism on a molecular level, a series of molecular dynamics (MD) simulations were performed. Two polymer models comprising WPM II and PCA/AP1.6 (see Fig. 4a and Supplementary Fig. 27) were constructed. The water dissociation energy barrier between the hydration layer and polymers was estimated using the water absorption energy. The water-dissociation energy barrier of WPM II reaches 3.00 kJ mol^−1^, 47% higher than that of PCA/AP1.6 (2.04 kJ mol^−1^), indicating that water molecules combine with WPM II MF to form a strong hydration layer that prevents the adhesion of oil molecules (see Fig. 4b and Supplementary Table 6). Furthermore, the dynamic characteristics of the diffusion coefficient and the residence time of water molecules in the hydration layer on the membrane surface are presented in Fig. 4c, d. The water molecular diffusion coefficients and residence time of water molecules in the hydration layer of WPM II and PCA/AP1.6 are 1.95 and 3.84 m^2^/s and 8.22 and 4.5 ps, respectively. The lower diffusion coefficient and higher residence time of water in WPM II certifies that the water molecules are bound more tightly to WPM II. On the other hand, the surface roughness of each membrane is closely related to crude oil fouling resistance. The surface roughness of the PCA/AP MFs is greatly improved when compared to that of the pristine MF, whereas the roughness of the WPM MF shows a decreasing trend when compared to that of the corresponding PCA/AP MFs (see Supplementary Fig. 28). The results of surface roughness show that with the deepening of the wrinkling mode, the roughness of the WPM MFs decreases (see Fig. 4e–g). This strategy of first increasing the roughness to enhance the hydrophilicity and then reducing the integrant roughness to lower the risk of foulant deposition on the membrane-surface gullies greatly reinforces the viscous crude oil antifouling performance of the WPM II MF. The surface free energies of the different membranes were calculated using the OWRK method (see Supplementary Table 7). The surface free energy of the WPM II MF is 88.23 mN/m, which is higher than that of PCA/AP1.6 (84.28 mN/m) and pristine MF (31 mN/m). Hence, the synergy of the enhanced hydration layer and sufficient steric hindrance properties establishes the PFP mechanism: the coating of smooth hydrophilic microparticles increases the hydrophilicity and surface roughness, forming a hydration layer to establish primary fouling resistance. Then, wrinkled patterns outlined on the smooth microparticle surface appropriately reduce the surface roughness, which not only maintains the superhydrophilicity but also reduces the oil droplet deposition on the membrane surface. The wrinkled patterns can improve the contact area between the water molecules and the membrane surface, further generating a dense hydration layer and increasing the steric hindrance interactions between the membrane surface and oil molecules. Finally, the dense hydration layer and steric hindrance complement each other to inhibit the initial adsorption, migration, and deposition of crude oil on the surface, regardless of the viscosity, achieving the purpose of resisting migratory crude oil fouling.

**Fig. 5:**
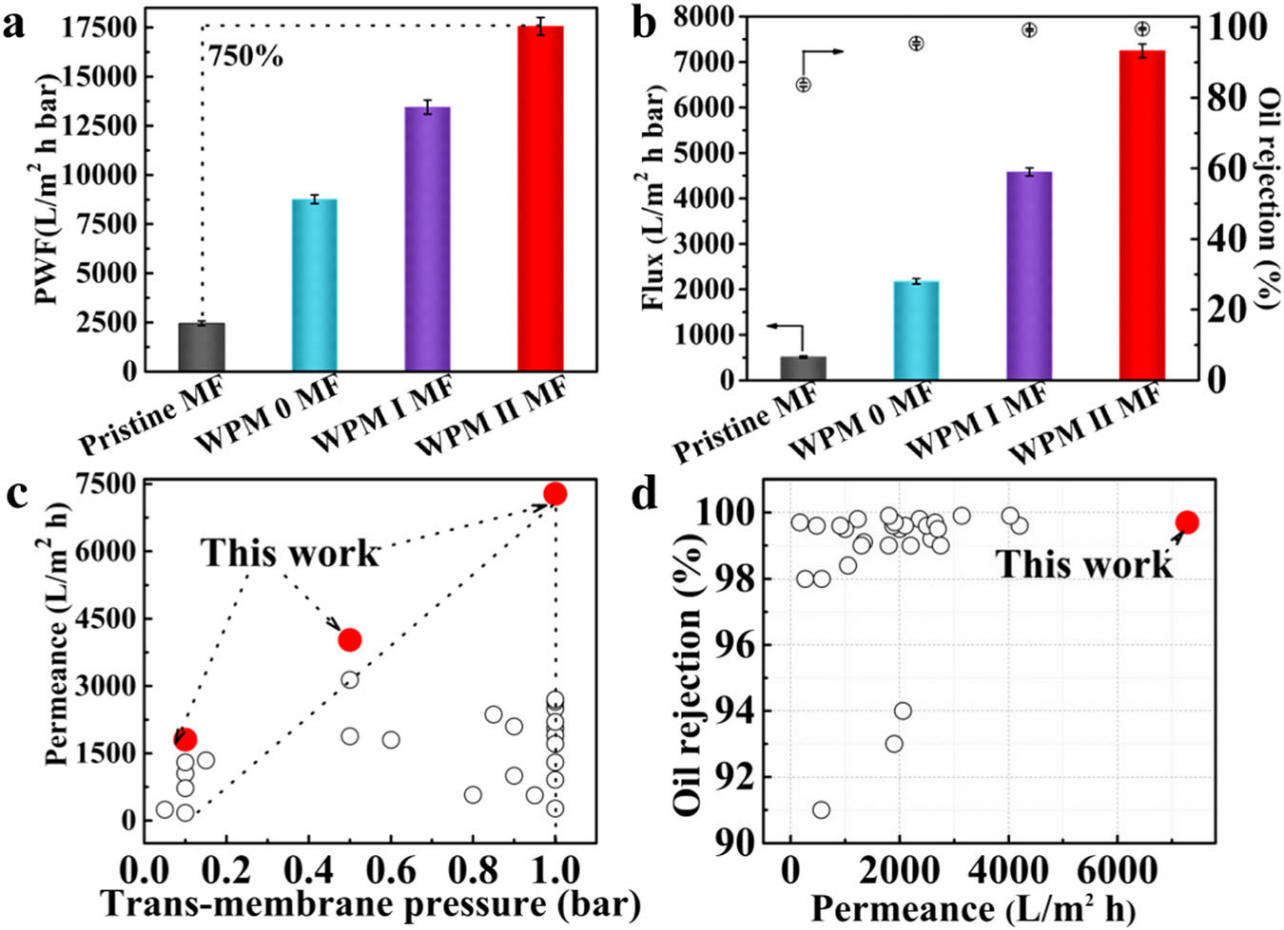
Performance of membranes for oil/water separation. **a** PWF of membranes. Error bars represent the standard deviations obtained from three membranes. PWF Pure water flux. Error bars represent the standard deviations obtained from three membranes. **b** Flux and separation efficiency of membranes. Error bars represent the standard deviations obtained from three membranes. **c**, **d** Comparison of the separation performances of the WPM II MF in this study and the state-of-the-art membranes reported in the literature.

To explore the effects of this PFP mechanism on oil/water separation applications, we tested the crude oil/water emulsion separation performance of the different membranes (see Fig. 5a, b and Supplementary Figs. 29–35). The pure water flux (PWF) of the WPM II MF reaches 17555.51 L m^−2^ h^−1^ bar, a 790% improvement when compared to that of commercial pristine MF (2000 L  m^−2^ h^−1^ bar) (see Supplementary Movie 4). Then, the flux of the WPM II MF reaches 7247 L m^−2^ h^−1^ bar, 1300% higher than that of the pristine MF (517 L m^−2^  h^−1^ bar) for crude oil in water (C/W) emulsions under 1 bar of dead-end filtration. The separation efficiency of the WPM II MF is 99.6% for crude oil in water (C/W) emulsions (see Supplementary Movie 5). This flux improvement is mainly because the wrinkled patterns on the smooth hydrophilic microparticles do not reduce the pore size of the membrane and optimize the contact area between the water and the membrane surface. These fluxes of the WPM II MF are maintained even after four rounds of repeated testing, demonstrating good recyclability for emulsion separation (see Supplementary Movie 6). WPM II MF can achieve crude oil/water emulsion separation mainly owing to the typical capillary effect of the hydrophilic pores (ΔP < 0)^42^. The spreading of the water phase on the WMP II MF surface occurs quickly, spontaneously forming a water layer. Coupled with a UOCA greater than 165 °, the WPM II MF possesses good oil prevention ability. In this case, demulsification occurs as the oil-in-water (O/W) emulsion contacts the membrane surface (see Supplementary Fig. 36)^43^, agglomerating water droplets for transportation across the WPM II MF while restricting the transportation of oil drops, achieving O/W emulsion separation. We tested the separation properties of the mixtures of water and viscous crude oil of the WPM II MF, and our membranes have potential practical applications for treating crude oil sewage (see Supplemental Fig. 37). Such separation performances were upheld under various operating conditions: acidic, alkaline, or saline (see Supplementary Figs. 38, 39)^44^. Owing to the PFP mechanism, the O/W emulsion separation performance of the WPM II MF developed here outperformed that of most other membranes (see Fig. 5c, d and Supplementary Table 9). The flux of the WPM II MF in Supplementary Fig. 40 shows that the separation flux can still be maintained above 2000 L m^−2^ h^−1^ bar under 1 bar of cross-flow filtration after 190 h of operation, indicating that the WPM II MF is capable of stable long-term separation performance.

### Thickness of the hydration layer dominated by the PFP mechanism

**Fig. 6:**
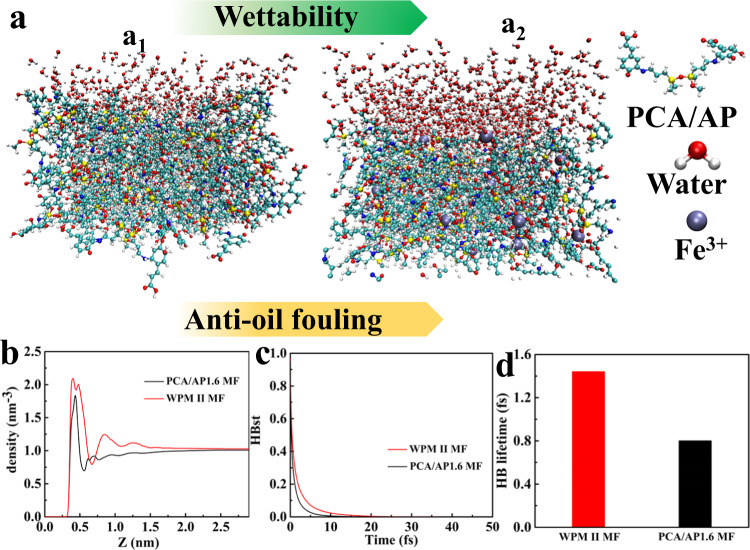
Hydration layer dominated by the PFP mechanism. **a** MD simulation snapshots showing **a**_**1**_ PCA/AP1.6 MF and **a**_**2**_ WPM II MF layers and water molecules that are within 3.4 Å above their surfaces. From left to right, the hydration states of these layers and HB strength increase, which is characterized by the water-PCA/AP1.6 MF or WPM II MF radial distribution functions. HB: Hydrogen bonds. **b** Density distribution of water molecules. **c** Continuous time correlation function of HB between interfacial water molecules and the membrane surface. **d** HB lifetime between interfacial water molecules and the membrane surface.

The crude oil antifouling performance of the WPM II MF is a consequence of its hydration states dominated by the PFP mechanism, that is, the interactions between the membrane surface and water molecules from the above antifouling experiments and water absorption energy. The thickness of the hydration layer and the strength of the HB determine the crude oil antifouling ability. Thus, classic MD simulations were performed to investigate the microstructure and HB properties at the interface of water and the membrane surface slab from the atomic level (see Fig. 6a). The water/membrane surface interactions were analyzed by the radial distribution functions (RDFs) of the oxygen of water with the surface. The RDFs indicate three distinct peaks associated with the first, second, and third solvation layers of water near the interface (see Fig. 6b). Water molecules are absorbed layer by layer with relatively weak cohesion between adjacent layers. In the RDFs, the first sharp peak reveals that the first water layer can adhere closely and strongly to all membrane surfaces. The peak height of the water molecular density on the WPM II MF surface is slightly higher than that on the PCA/AP1.6 MF surface. Combined with the experimental results of the antifouling performance of low-viscosity oil, both the traditional PRF mechanism and the PFP mechanism exhibit certain anti-oil-fouling performance. However, compared with the relatively wide and high second peak of water molecular density on WPM II MF, the second water layer of PCA/AP1.6 MF is weakly attached to the surface. Furthermore, the third water layer of the WPM II MF surface is much higher than the second water layer of PCA/AP1.6 MF, resulting in the membrane modified by the PFP mechanism strategy possessing strong resistance to viscous crude oil fouling. Specifically, the thickness of the water layer on the surface of WPM II and PCA/AP1.6 MF is 0.36 and 0.25 nm, respectively. In addition, the continuous time correlation function (CTCF) of HB between the interfacial water molecules and membrane surface was analyzed using the trajectory collected by HB dynamics. The CTCF of HB was used to represent the process of HB formation to fracture and could be used to characterize the strength of the HB. The slower the decay of the CTCF curve of the HB, the stronger the strength of the HB. Figure 6c shows that the decay rate of the CTCF of HB between the interfacial water molecules and WPM II MF surface is smaller than that of the PCA/AP1.6 MF surface, which indicates that the HB strength between the interfacial water molecules and WPM II MF surface is greater than that between the interfacial water molecules and the PCA/AP1.6 MF surface. In addition, the lifetime of HB between the interfacial water molecules and WPM II MF is 1.5 fs, which is 1.9 times that of PCA/AP1.6 MF (0.8 fs) (see Fig. 6d). Consequently, the thickness of the hydration layer and HB strength dominated by the PFP mechanism can effectively prevent fouling from viscous crude oil and expand the application of the polymer separation membrane in the field of crude oil spill treatment and oil/water separation. In addition, the *dahlia*-leaves-inspired hierarchical structure and HB strength contribute to stronger mechanical properties and thermal stability (see Supplementary Figs. 36, 37).

## Discussion

To overcome the bottleneck of the conventional PRF mechanism in terms of migratory viscous crude oil fouling, we incorporated superhydrophilicity with hierarchical architecture across the micro/nanoscales to create a PFP mechanism based on the synergy between the hydration layer and sufficient steric hindrance interactions. Wrinkled patterns outlined on the smooth microparticle surfaces could improve the contact area between water molecules and the membrane surface, further generating a dense hydration layer and increasing the steric hindrance interactions between the membrane surface and oil molecules. Finally, the hydration layer and steric hindrance complemented each other to inhibit the initial adsorption, migration, and deposition of crude oil on the surface, regardless of the viscosity, and achieved the purpose of resisting migratory crude oil fouling. Our superhydrophilic membrane demonstrated a water permeance of 7247 L m^−2^ h^−1^ bar and precise crude oil/water emulsion separation. Researchers can extend this concept of introducing wrinkled surface patterns to microdroplet control, flexible wearable devices, biochemical protection, actuators, and other fields, further expanding the application of superwettability.

## Methods

### Material preparation

Commercial poly (vinylidene fluoride) (PVDF) microfiltration (MF) flat membranes were obtained from Beijing Qichao (China). Caffeic acid (CA), (3-aminopropyl) triethoxysilane (APTES), iron (III) chloride hexahydrate (FeCl_3_·6H_2_O), oil red O, tris (hydroxymethyl) aminomethane (Tris) and hydrochloric acid (HCl) were purchased from Aladdin (China). Ethanol, dichloromethane, and toluene were obtained from Yuanli Chemical Co., Ltd. (China). Light crude oil with low viscosity (API Gravity > 20°) and viscous crude oil (API Gravity: 10–20°) were obtained by SINOPEC SABIC Tianjin Petrochemical Co., Ltd. (China). All chemicals can be used directly without further purification.

### Synthesis of PCA/AP microparticles

First, 0.2 g of caffeic acid (CA) was dissolved in 100 mL of Tris-HCl buffer solution (pH = 8.5) and then stirred to a homogeneous solution. Second, three different amounts of (3-aminopropyl) triethoxysilane (APTES) (0.4 g, 0.8 g, and 1.6 g, respectively) were added and stirred for 12 h. Third, the suspension was centrifuged for 10 min to remove the supernatant, and the obtained product was washed with deionized water three times. Finally, the resulting PCA/AP0.4, PCA/AP0.8, and PCA/AP1.6 microparticles were reserved for future use.

### Synthesis of wrinkled microparticles

The as-prepared PCA/AP0.4, PCA/AP0.8, and PCA/AP1.6 microparticles were dissolved into 100 mL of FeCl_3_·6H_2_O (1 mg/ml) solution, and microparticles with different morphologies were fabricated. The obtained products were centrifuged for 5 min and washed with ethanol three times. Then, they were dried in an oven at 60 °C for 12 h.

### Preparation of the modified membranes

The whole modified membranes were prepared by the surface coating method. The pristine MF membrane was dipped in ethanol solution for 3 h before use, rinsed with deionized water, and then soaked in deionized water for 3 h again. Subsequently, the membrane was immersed in 100 ml Tris-HCl buffer solution (pH = 8.5) of CA at a concentration of 2 mg/ml in a Petri dish for 1 h to achieve sufficient PCA adsorption on the membrane surface. Then, 0.4 g, 0.8 g, and 1.6 g APTES in 5 ml ethanol was added to the Petri dish and then shaken in a water bath for 24 h. Then, the PCA/AP membranes were washed for several minutes with deionized water to remove residual solutions. The as-prepared membranes were submerged into 100 ml FeCl_3_·6H_2_O solution (1 mg/ml) for 1 h, and the resulting membranes were fabricated (Supplementary Table 1). Finally, the obtained membranes were flushed with deionized water and dried in a vacuum oven at 30 °C.

### Estimation of excess stress

In essence, wrinkle is caused by instability by external stimulation of spherical film/substrate systems, and the surface yield deformation of the system is spontaneously generated to regain mechanical equilibrium and release excessive internal stress^25^. The wrinkled patterns are engendered spontaneously mainly due to the mismatch in the coefficient of thermal expansion between the Fe films and the PCA/AP microparticles. The compressive hoop stress *σ*_*f*_ in the film was estimated by the following equation quantitatively characterized by the Swift-Hohenberg theory^26^:

6 $${\sigma }_{f}=\frac{E{E}_{s}(3{R}^{3}+3{R}^{2}{{{{{\rm{h}}}}}}+3R{h}^{2}+{h}^{3})({\alpha }_{s}-\alpha )\varDelta T}{3{E}_{s}{R}^{3}(1-V)+{E}_{s}(1+v)(3{R}^{2}{{{{{\rm{h}}}}}}+3R{h}^{2}+{h}^{3})+3E(1-2{V}_{S}){{{{{\rm{h}}}}}}(3{R}^{2}+3R{{{{{\rm{h}}}}}}+{h}^{2})}$$

where *R* represents the microparticle radius, h represents the film thickness, *E* represents the elastic modulus, *ΔT* represents the actual temperature change during deformation, *α* represents the coefficient of thermal expansion, *ν* represents Poisson’s ratio, and subscripts s and f represent the substrate and the film, respectively. Meanwhile, the critical wrinkling stress σ_c_ for spherical systems was expressed as the following equation:

7 $${{{{{\rm{\sigma }}}}}}=(1+{\varOmega }^{2}){\sigma }_{c}$$

where *Ω* represents a dimensionless curvature parameter, *σ*_*c*_ represents the critical wrinkling stress for planar systems, and *Ω* is given by the following equation:

8 $$\varOmega=\root {2} \of {(1-{\upsilon }_{f}^{2})}({{h}}/{{R}}){({\bar{E}}_{f}/2{\bar{E}}_{s})}^{2/3}$$

*σ*_*c*_ is given by the following equation:

9 $${\sigma }_{c}=\frac{{\bar{E}}_{f}}{4}{\left(\frac{3{\bar{E}}_{s}}{{\bar{E}}_{f}}\right)}^{2/3}$$

where $\bar{E}$ represents the plane strain modulus, expressed as the following equation:

10 $$\bar{E}=\frac{E}{1-{\upsilon }^{2}}$$

The excess stress is given by the following equation:

11 $$\sum=\frac{{\sigma }_{f}}{\sigma }-1$$

### Formation of wrinkled patterns

To quantitatively describe curvature-related wrinkled patterns, Dunkel et al. recently proposed the generalized Swift-Hohenberg theory^27^. The selection criteria for wrinkled patterns are as follows:

12 $${{{{{\rm{Unwrinkled}}}}}}{\!}:\sum \le 0$$

13 $${{{{{\rm{Dimple}}}}}}\,{{{{{\rm{pattern}}}}}}{\!}:-\frac{\rho }{20}{\left(\frac{h}{R}\right)}^{2} < \sum \, < \,\rho {\left(\frac{h}{R}\right)}^{2}$$

14 $${{{{{\rm{Labyrinth}}}}}}\,{{{{{\rm{pattern}}}}}}{\!}:4\rho {\left(\frac{h}{R}\right)}^{2} < \sum \, < \,20\rho {\left(\frac{h}{R}\right)}^{2}$$

15 $${{{{{\rm{Herringbone}}}}}}\,{{{{{\rm{pattern}}}}}}{\!}:\sum \, > \,20\rho {\left(\frac{h}{R}\right)}^{2}$$

where ρ represents a fitting parameter expressed by $\rho={[{(\frac{3{E}_{S}}{{E}_{f}})}^{1/3}(1-\upsilon )\tilde{c}]}^{-2}$, the parameter $\tilde{c}$, as a function of *E*_*f*_/*E*_*s*_ and *ν*, which predicates the phase transition boundaries hinge on the material properties.

### Characterizations

The morphological images of each membrane were analyzed by scanning electron microscopy (SEM, Nanosem 430) with an accelerating voltage of 20 kV attached to an energy-dispersive spectrometer (EDXS) (Hitachi SU8020). The surface roughness of all membranes was measured by atomic force microscopy (AFM, Bruker Dimension Fastscan) with Nanoscope5 electronics. The tips were from Nanosensors with a nominal spring constant of 7 N/m and a typical radius of less than 5 nm. Confocal laser scanning microscopy (CLSM) Confocal laser scanning microscopy (CLSM, Olympus 3000) was used to analyse the structure of the membrane surface. Three samples for each membrane were prepared, and three points were selected on each sample for parallel testing. The pore size distribution of the membranes was measured with a capillary flow porometer (Beijing Bayside Technology Apparatus, 3H-2000 PB). The size distribution of microparticles and oil droplets in the O/W emulsion and filtration were determined by dynamic light scattering measurement (DLS, Malven ZS90). The porosity (*ε*) of the membrane was calculated by the gravimetric method using the following equation:

16 $$\varepsilon=\frac{{W}_{{{{{{\rm{wet}}}}}}}-{W}_{{{{{{\rm{dry}}}}}}}}{\rho {{{{{\rm{A}}}}}}{\delta }_{0}}\times 100\%$$

where *W*_*wet*_ (g) and *W*_*dry*_ (g) are the membrane weights at wet and dry, respectively, *ρ* (g cm^−3^) is the water density at room temperature, *δ*_*0*_ (cm) is the average membrane thickness, and *A* (cm^2^) is the effective membrane area. Fourier transform infrared spectroscopy (FTIR) was performed on a spectrometer (Nicolet, Nicolet-560). X-ray photoelectron spectroscopy (XPS) was performed using an AXIS ULTRA DLD spectrometer (Shimadzu, Japan) with a monochromatized Al Kα X-ray source (1486.6 eV photons). FTIR and XPS were analyzed to investigate the chemical components of all membranes. The wettability of these membranes was evaluated by a contact angle meter (Kruss Gmbh, DSA 100) at room temperature. All measurements were taken three times to ensure their accuracy. The size distribution Optical images of the O/W emulsion before and after separation were obtained from a microscope (BX51TF Instec H601) by dropping the solution on a wafer. The underwater anti-crude oil-adhesion property of the membrane was investigated based on a surface tension detector (Kruss Gmbh, K100). The adhesion work can be defined as the work required to separate oil from the surface in water, which is a manifestation of the adhesive force of oil on the membrane surface underwater and can reflect the adhesion strength between oil and the membrane surface. The adhesion work underwater is calculated by W = γ (1 + cos θ), where θ is the UOCA at the membrane surface or silicon wafer and γ is the interfacial tension between water and crude oil. To further investigate the hydration layer, we performed a free-falling experiment of oil droplets in water. When oil droplets go free-falling in water and touch the membrane surface, their force analysis on a membrane surface underwater can be based on the energy conservation Equation. 1/2*mν*^2^ = (*G*-*F*_*b*_) *h*^2^ + *J*_*e*_ + *J*_*a*_, where m is the oil droplet mass (the oil drop mass is 0.0318 g), *ν* is the oil droplet rebound, *G* is the oil droplet gravity, *h*_*1*_ is the initial height of the oil droplet (initial height is 5.5 cm), *h*_*2*_ is the rebound height of the oil droplet, *F*_*b*_ is the buoyancy of the oil droplet, *F*_*b*_ = *ρ*g4/3π*r*^3^, where *r is* the radius of the oil droplet, *F*_*e*_ is the elastic force of the oil droplet, *J*_*e*_ is the work of *F*_*e*_ as the oil droplet contacts the membrane surface, *F*_*a*_ is the adhesive force of the oil droplet, *θ* is the UOCA, and *J*_*a*_ is the work of *F*_*a*_ as the oil droplet contacts the membrane surface. Thermogravimetric analyses (TGA) were measured on a MettlerToledoSDTA-854 TGA/DSC system under nitrogen (100 mL min^–1^) in the temperature range from room temperature to 800 °C under a N_2_ atmosphere at a heating rate of 10 °C min^–1^. After the wrinkled pattern is formed on the surface of the microparticles, the surface area of the membrane will change, which will affect the contact area between the surface of the modified membrane and the water molecules. Therefore, we choose to calculate the changes in the surface area and volume of the microparticle through 3D MAX software simulation.

### Simulation methods

Density functional theory (DFT) is a commonly used computational tool because of its high degree of predictive power. DFT calculations are performed using Gaussian 16 quantum chemistry software. All the molecules are preoptimized at the B3LYP-D3(BJ)/6-31 G* level. Here, the self-consistent reaction field (SCRF) theory of SMD-flavor was adopted^45^. Then, the configuration of the complex was optimized at the B3LYP/6-31 G(d) level. Grimme’s D3BJ dispersion was used to describe the intermolecular interactions. The single point energy and binding energies were calculated at the B3LYP/6-311 G(d) level based on the following equation: Binding energy = E_complex_ – (E_fragment1_ + E_fragment2_)

Molecular dynamics (MD) simulations were performed using the GROMACS software package (version 4.0.5). In the simulation, the Gromos53a6 force field is adopted. The force field parameters for PCA/AP and WPM II MF used in this work are listed in Table 5 in the supporting information. Two hydrogel polymers with a size of 4 nm*4 nm*10 nm is constructed as system I for PCA/AP1.6 MF and system II for WPM II MF, respectively. These systems are initialized by minimizing the energies of the initial configurations using the steepest descent method. Then, a 10 ns MD simulation under canonical ensemble (NVT) is carried out.

MD simulations were carried out to investigate the microstructure and hydrogen bond (HB) properties at the interface of water and inorganic molecule slabs at the atomic level. Here, two kinds of molecule slabs were constructed: one consisting of 90 PCA/AP molecules and the other consisting of 90 PCA/AP molecules and 30 Fe^3+^ ions. Both were compressed to form the molecule slabs. Then, 6000 water molecules were randomly inserted on the molecule slabs to construct the interface system by the software of PACKMOL. The GROMOS54a7 force field^2^ was employed to describe the behavior of the molecules. The molecular force field consists of nonbonded and bonded interactions. The nonbonded interaction contains van deer Waals (vdW) and electrostatic interactions, which are described by equation 17, 18, respectively.

17 $${E}_{{{{{{\rm{LJ}}}}}}}({r}_{{{{{{\rm{ij}}}}}}})=4{\varepsilon }_{{{{{{\rm{ij}}}}}}}\left\{{\left(\frac{{\varepsilon }_{{{{{{\rm{ij}}}}}}}}{{r}_{{{{{{\rm{ij}}}}}}}}\right)}^{12}-{\left(\frac{{\varepsilon }_{{{{{{\rm{ij}}}}}}}}{{r}_{{{{{{\rm{ij}}}}}}}}\right)}^{6}\right\}$$

18 $${E}_{c}({r}_{{{{{{\rm{ij}}}}}}})=\frac{{q}_{i}{q}_{j}}{4\pi {\varepsilon }_{0}{\varepsilon }_{r}{r}_{{{{{{\rm{ij}}}}}}}}$$

For different kinds of atoms, the Lorentz-Berthelot mix rules were adopted for vdW interactions. The cutoff distance of vdW and electronic interactions was set to 1.2 nm, and the particle mesh Ewald (PME) method was employed to calculate long-range electrostatic interactions.

19 $${\sigma }_{{{{{{\rm{ij}}}}}}}=\frac{1}{2}({\sigma }_{{{{{{\rm{ii}}}}}}}+{\sigma }_{{{{{{\rm{jj}}}}}}});{\varepsilon }_{{{{{{\rm{ij}}}}}}}={({\varepsilon }_{{{{{{\rm{ii}}}}}}}\ast {\varepsilon }_{{{{{{\rm{jj}}}}}}})}^{\frac{1}{2}}$$

### O/W emulsion separation performance

Oil-in-water (O/W) emulsion filtration experiments were assessed, and the separation performance of all membranes was compared: 10 ml of oil (crude oil, toluene, petroleum ether, 1,2-dichloroethane and silicone oil) and 0.01 g of Tween 80 were added to 490 mL of water with vigorous stirring for 3 h to acquire the crude oil-in-water (C/W) emulsion, toluene-in-water (T/W) emulsion, petroleum ether-in-water (P/W) emulsion, 1,2-dichloroethane (D/W) emulsion and silicone oil-in-water (Si/W) emulsion. The separation performance test mainly includes two methods. One is a dead-end filtration device (vacuum filtration equipment), which is mainly suitable for short-term separation flux and oil rejection tests and is a common laboratory preliminary test method for oil/water emulsion separation. The other is a cross-flow filtration device, which is often used for long-term oil/water emulsion separation performance tests after dead-end filtration tests. Thus, we tested the oil/water emulsion separation of all membranes by vacuum filtration equipment with a constant transmembrane pressure of 1 bar within 700 minutes and recorded the flux and oil rejection. The water flux of each membrane was determined by the following equation:

20 $${{{{{\rm{J}}}}}}=\frac{\varDelta {V}}{{A}\varDelta {t}}$$

where *∆V* (L) is the permeate volume, *A* (m^2^) is the effective area (12.56 × 10^−4^ m^2^) of the membrane, and *Δt* (h) is the filtration time. During the filtration process, the filtration volume was constant at 300 ml, and the filtration time was recorded. Each membrane was tested five times. Moreover, the long-term water permeation of the prepared membranes was characterized through a cross-flow filtration setup. Samples were placed in a flat module with an effective surface area of 35 cm^2^. All experiments were carried out at 1.0 bar and 25 °C with a Reynolds number of approximately 2400. The flux of WPM II MF was evaluated for long-term operation stability within 190 h for crude oil/water emulsion separation. The flux included four stages: (1) water flux for crude oil/water emulsion separation was measured for 48 h. After that, the membrane was washed in deionized water for 1 h; (2) the subsequent flux was carried out for another 48 h and washed in deionized water for 1 h; (3) and (4) the above experimental steps (1) and (2) were repeated. The separation flux was recorded at one-hour intervals.

The oil rejection of the filtration process is calculated by the following equation:

21 $${{{{{\rm{R}}}}}}={\left(1-\frac{{C}_{1}}{{C}_{0}}\right)}\times 100\%$$

where R is the separation efficiency and *C*_*0*_ and *C*_*1*_ are the oil concentrations of the emulsion before and after filtration and tested with a UV spectrophotometer, respectively.

### Anti-oil fouling performance

The anti-oil fouling capacity of the membranes was evaluated by the flux recovery ratio (FRR, %). The flux and oil rejection of all membranes by dead-end filtration were investigated by vacuum filtration equipment with a constant transmembrane pressure of 1.0 bar. We used pure water filtration first and recorded the flux at one-minute intervals until the flux was stable and then tested the separation of the oil/water emulsion. Thus, the separation performance of the test was more stable. The initial pure water flux was recorded as *J*_*w0*_. Subsequently, C/W emulsion separation was tested at a filtration pressure of 1 bar until the flux was stable, and the filtration flux (*J*_*w1*_) was recorded. Then, the membrane was cleaned with deionized water for several minutes, and the pure water filtration operation was repeated, recording the stable pure water flux (*J*_*w2*_). The C/W emulsion was placed in a filtration cell to conduct the second test cycle after the first test cycle, as described above. The FRR, total fouling ratio (R_t_), reversible ratio (R_r_) and irreversible fouling ratio (R_ir_) were used as indexes to evaluate the anti-oil fouling resistance of the membrane; the higher the FRR of the membrane, the lower the Rt of the membrane, revealing better anti-oil fouling of the membrane. They are calculated by the following equations:

22 $${{{{{\rm{FRR}}}}}}=\frac{{J}_{{{{{{\rm{w2}}}}}}}}{{J}_{{{{{{\rm{w0}}}}}}}}\times 100\%$$

23 $${R}_{t}=\left(1-\frac{{J}_{{{{{{\rm{w1}}}}}}}}{{J}_{{{{{{\rm{w0}}}}}}}}\right)\times 100\%$$

24 $${R}_{{{{{{\rm{ir}}}}}}}=\left(1-\frac{{J}_{{{{{{\rm{w2}}}}}}}}{{J}_{{{{{{\rm{w0}}}}}}}}\right)\times 100\%$$

25 $${R}_{r}=\frac{{J}_{{{{{{\rm{w2}}}}}}}-{J}_{{{{{{\rm{w1}}}}}}}}{{J}_{{{{{{\rm{w0}}}}}}}}\times 100\%$$

## Supplementary information

Supplementary Information

Supplementary Movie 1

Supplementary Movie 2

Supplementary Movie 3

Supplementary Movie 4

Supplementary Movie 5

Supplementary Movie 6

## Data Availability

Source data are provided in this paper. The data used in this study are presented in the text, Supplementary Information, and Source Data. Additional data and information are available from the corresponding author upon request. Source data are provided in this paper.

## Source data

Source Data
