# Supplementary material for: Surface manipulation for prevention of migratory viscous crude oil fouling in superhydrophilic membranes
Source: Nat Commun. 2023 May 9;14:2679. doi: 10.1038/s41467-023-38419-3 (PMC10169857; doi:10.1038/s41467-023-38419-3)
Supplement: Supplementary file 1 — Supplementary Information [file 41467_2023_38419_MOESM1_ESM.pdf]

Supplementary Information

Surface Manipulation for Prevention of Migratory Viscous Crude Oil Fouling in Superhydrophilic Membranes

Yuanyuan Zhao[a], Xiaobin Yang[a], Zhongjun Cheng[a], Cher Hon Lau[b], Jun Ma[c], Lu Shao[a]*

[a] MIIT Key Laboratory of Critical Materials Technology for New Energy Conversion and Storage, State Key Laboratory of Urban Water Resource and Environment, School of Chemistry and Chemical Engineering, Harbin Institute of Technology, Xidazhi 92, Harbin 150001, PR China

[b] School of Engineering, The University of Edinburgh, The King's Buildings, Mayfield Road, EH9 3JL, United Kingdom

[c] School of Environments; Harbin Institute of Technology; Harbin 150001, PR China

*e-mail: shaolu@hit.edu.cn

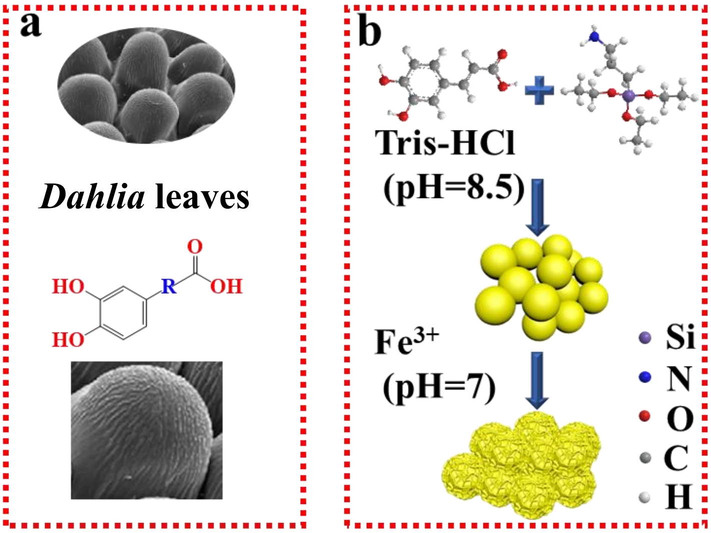

Supplementary Figure 1| **(a)** Structures of caffeoyl esters and flavonoids in *dahlia* leaves[1]; **(b)** Synthesis of wrinkled patterns on smooth microparticles via a two-step process: 1) Synthesizing smooth hydrophilic microparticles. 2) Creating cuticle-like nanostructures on microparticles surfaces via microparticles and Fe^{3+} coordination.

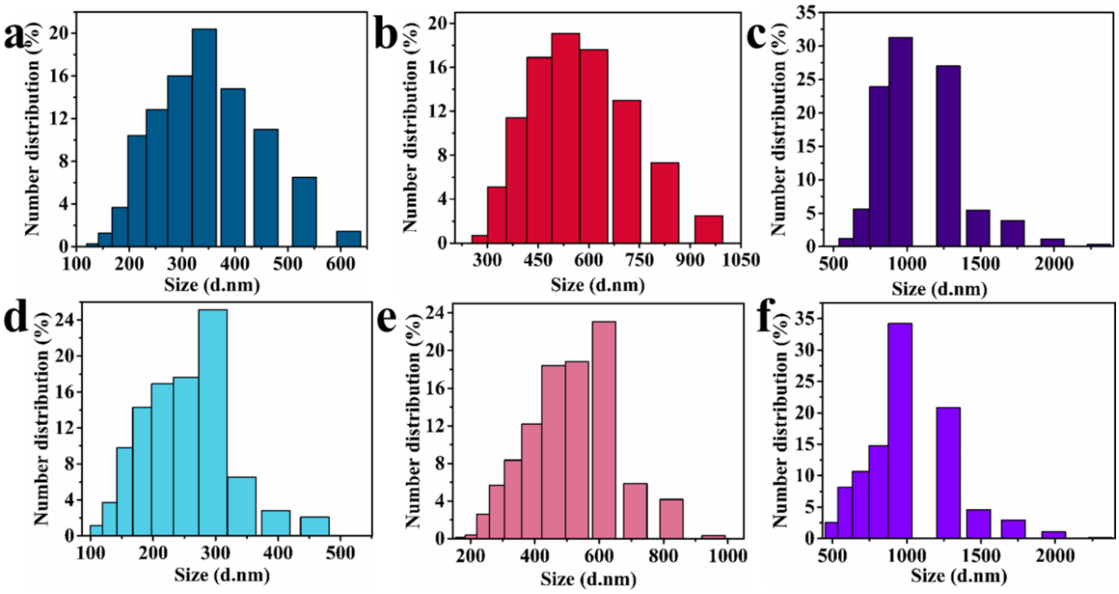

Supplementary Figure 2| The responding size distribution of microparticles of **(a)** PCA/AP0.4; **(b)** PCA/AP0.8; **(c)** PCA/AP1.6; **(d)** WPM 0; **(e)** WPM I; **(f)** WPM II.

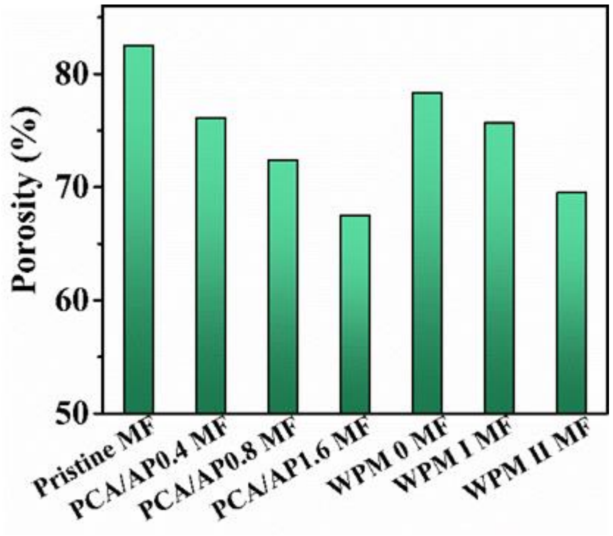

Supplementary Figure 3| (a) The porosity of the membranes.

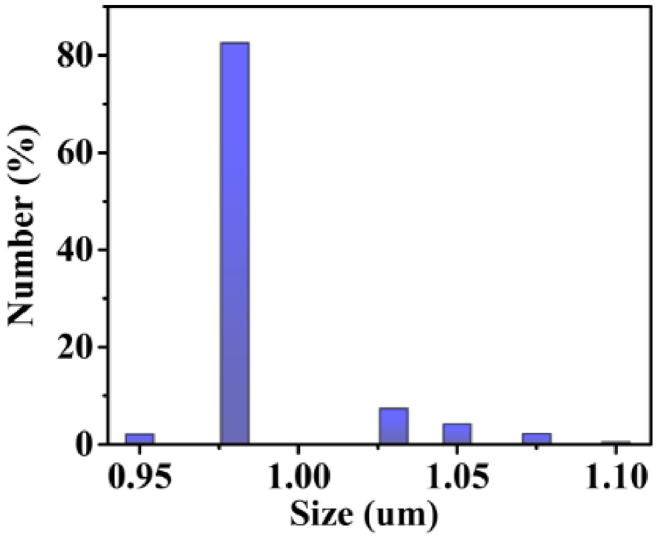

Supplementary Figure 4| The responding pore size distribution of the pristine MF.

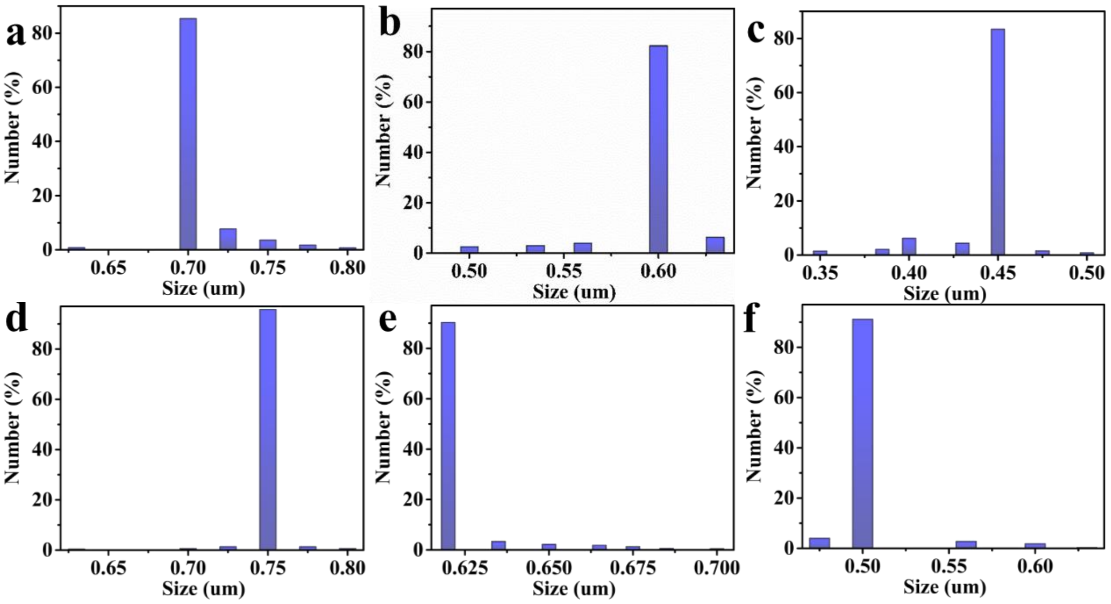

Supplementary Figure 5| The responding pore size distribution of (a) PCA/AP0.4; (b) PCA/AP0.8; (c) PCA/AP1.6; (d) WPM 0; (e) WPM I; (f) WPM II MF.

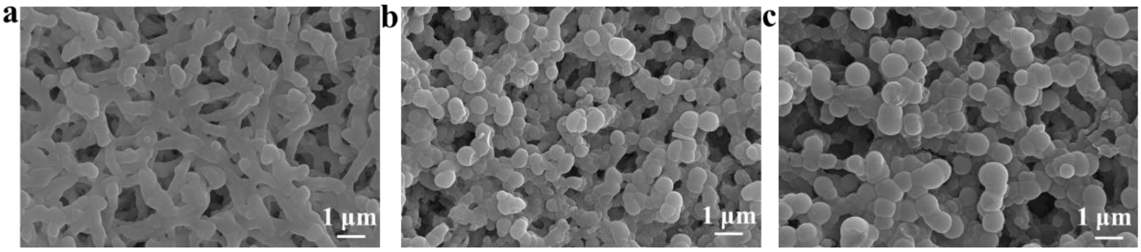

Supplementary Figure 6| SEM images of the surface of **(a)** PCA/AP0.4; **(b)** PCA/AP0.8; **(c)** PCA/AP1.6 MF.

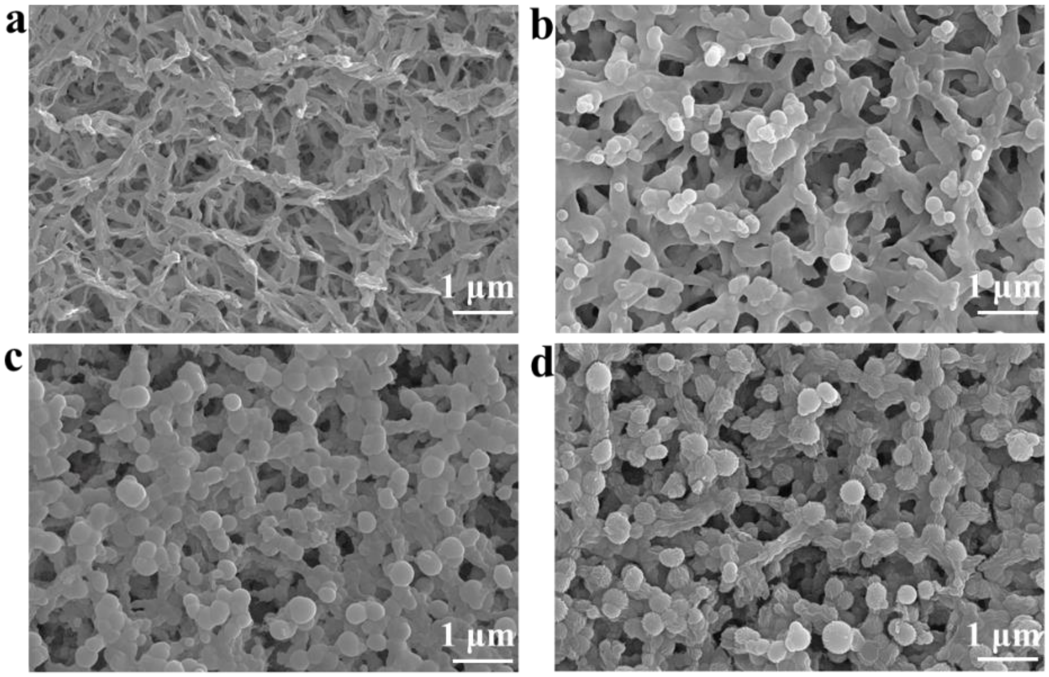

Supplementary Figure 7| (a-d) SEM images of the pristine MF, WPM 0, WPM I, and WPM II MF.

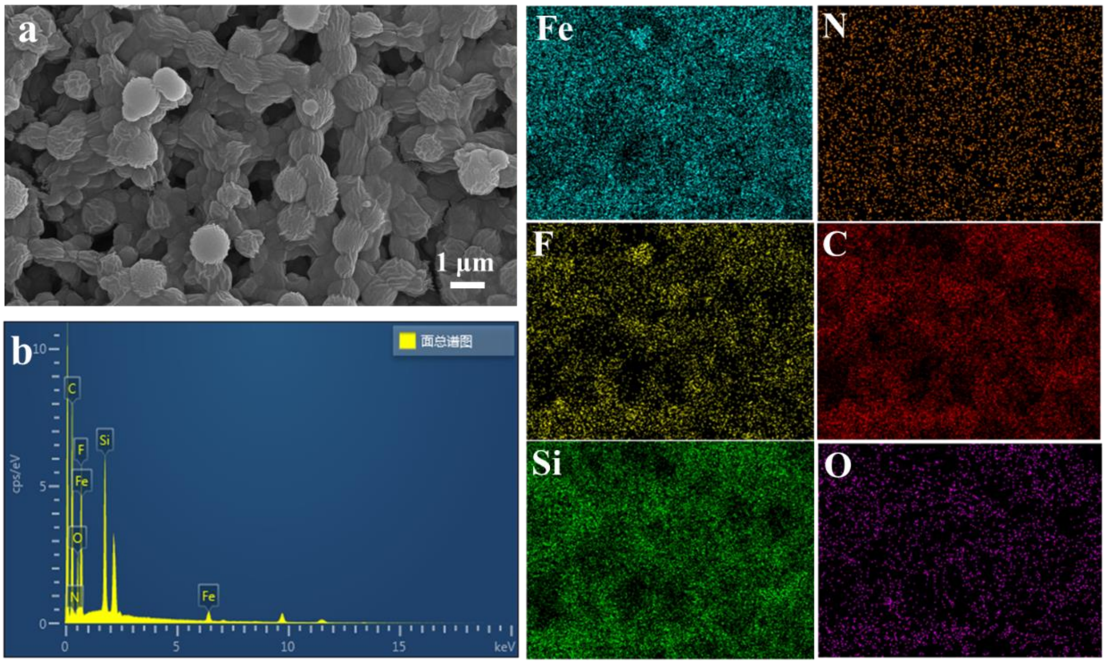

Supplementary Figure 8| (a) SEM images; **(b)** Corresponding EDS mapping of the WPM II MF.

Compared with the typical fibrous membrane pore structure of the pristine MF, the pore size and porosity of PCA/AP MFs decreased with the increase of the APTES content,

which was mainly due to the deposition of gradual increase PCA/AP microparticles on the surface of the membrane. While, the pore size and porosity of each WPM MFs had a weak upward trend compared to the corresponding PCA/AP MFs, which was mainly due to the deformation of the microparticles surface after Fe^{3+} modification. The pore size of WPM 0 MF (0.75 μm) increased 7.1% than that of PCA/AP0.4. The pore size of WPM I MF (0.625 μm) increased 4.1% than that of PCA/AP0.8 and the pore size of WPM II MF (0.5 μm) increased 11.1% than that of PCA/AP1.6. This varying degree of wrinkling reduced the space for microparticles to be coated on WPM MFs surface resulting in the pore size and porosity of WPM MFs would have a weak increase, which also can be confirmed by SEM image of each membrane in Supplementary Figs. 6 and 7. The results of SEM images and corresponding EDS mapping of the membrane in Supplementary Fig. 8 showed that we chose the synthesis process of the membranes has been shaken in a water bath to ensure more uniform distribution of microparticles on the membrane surface.

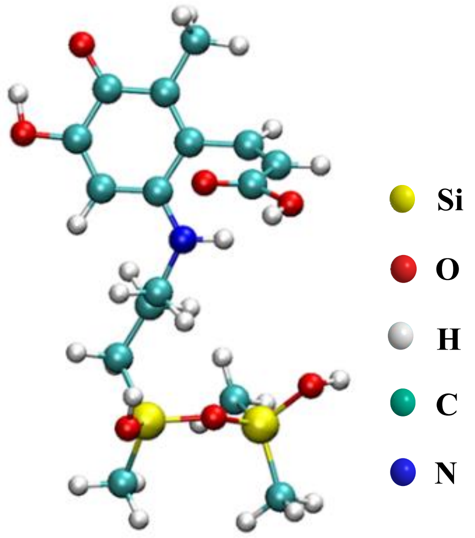

Supplementary Figure 9| Optimized geometry of PCA/AP.

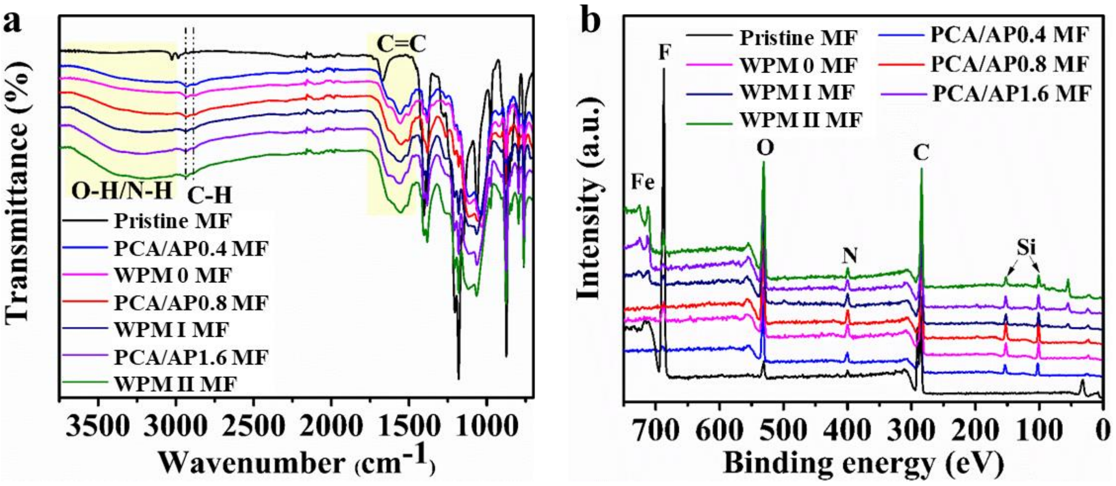

Supplementary Figure 10| (a) FTIR spectra and **(b)** XPS spectra of all membranes.

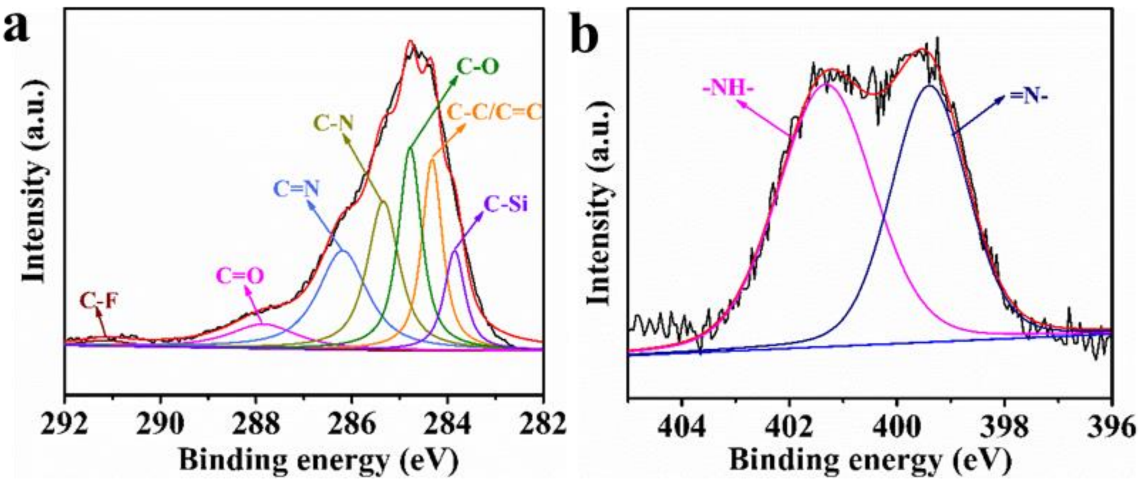

Supplementary Figure 11| The C 1s and N 1s spectrum of PCA/AP1.6 MF.

The wide absorption peak at 3000-3500 cm^{-1} were attributed to the stretching of O-H/N-H in the phenolic hydroxyl group/amino group of the modification membranes. The broad absorption band at 1650 cm^{-1} was assigned to benzene ring C=C of PCA, as well as, the new peaks at 1125 and 910 cm^{-1} were corresponding to Si-O-Si and Si-OH, respectively

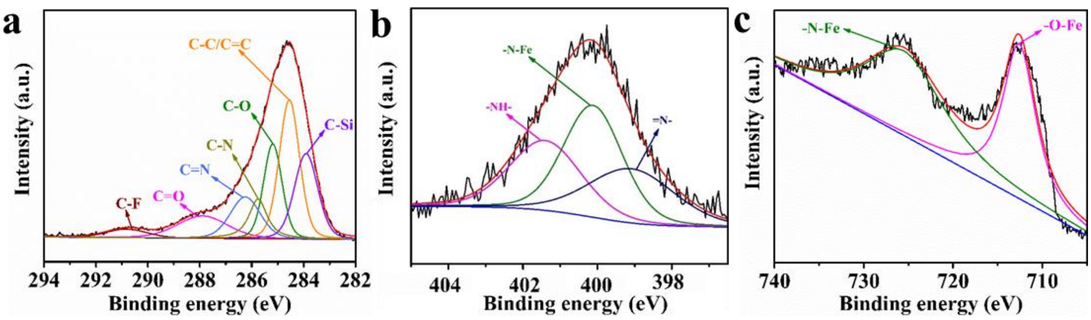

Supplementary Figure 12| The C 1s, N 1s and Fe 2p spectrum of WPM II MF[2].

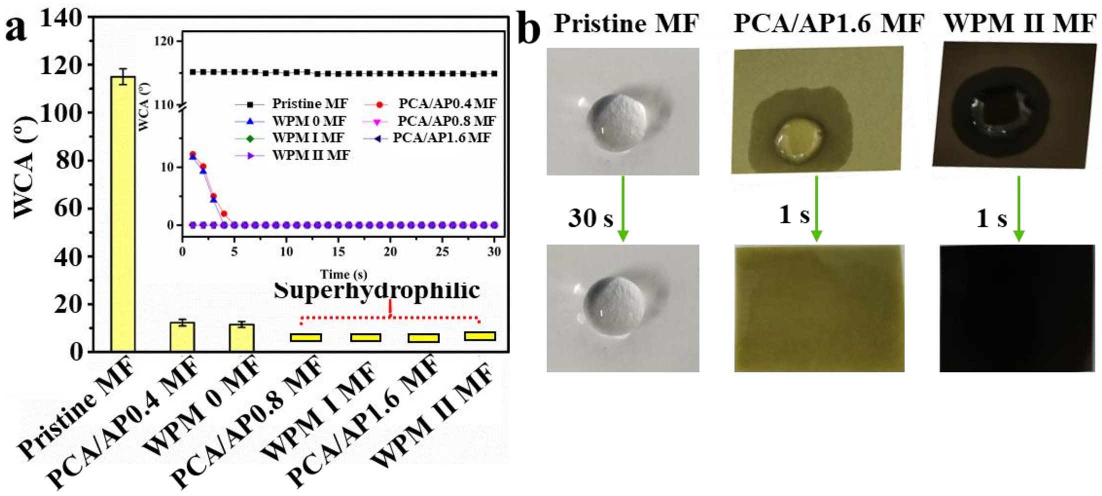

Supplementary Figure 13| (a) and **(b)** WCA and UOCA of different membranes. Error bars represent the standard deviations obtained from three membranes

Water contact angle (WCA) is comprehensively investigated the surface wettability of membranes. The pristine MF showed high hydrophobic with the initial WCA of 120 °, and water (30 μL) remained for 30 seconds without spreading on the surface of this membrane (see Supplementary Fig. 13). Diversely, thanks to the hydrophilic phenolic hydroxyl group/amino group, PCA/AP MFs were more affinity to water. PCA/AP0.8, PCA/AP1.6 MF and all the WPM MFs reached superhydrophilicity. These results showed the coating microparticles on the membrane surface endowed them with excellent affinity with water.

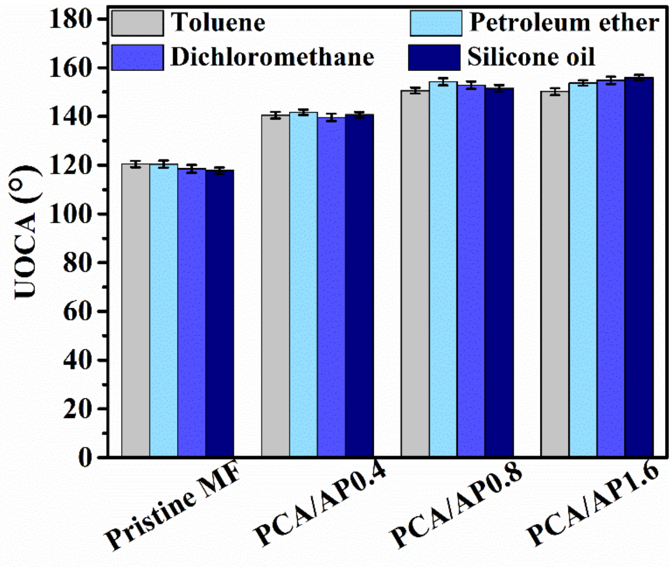

Supplementary Figure 14| The UOCA of PCA/AP MFs. Error bars represent the standard deviations obtained from three membranes

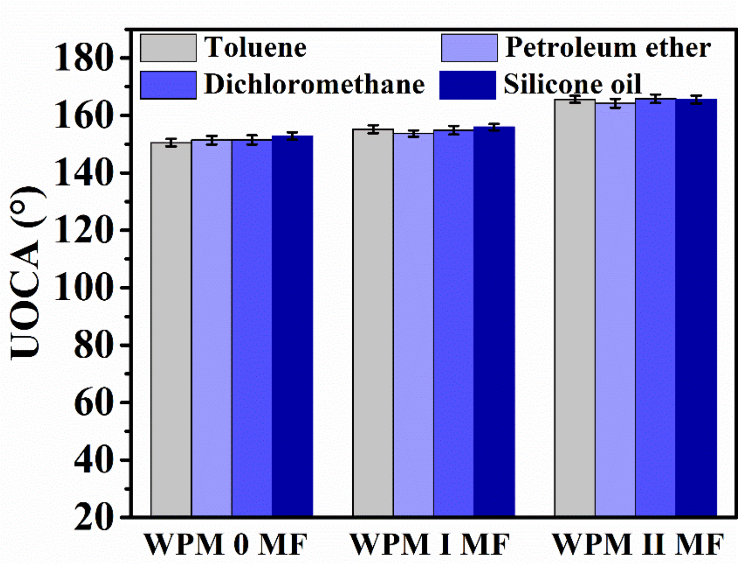

Supplementary Figure 15| The UOCA of WPM MFs. Error bars represent the standard deviations obtained from three membranes

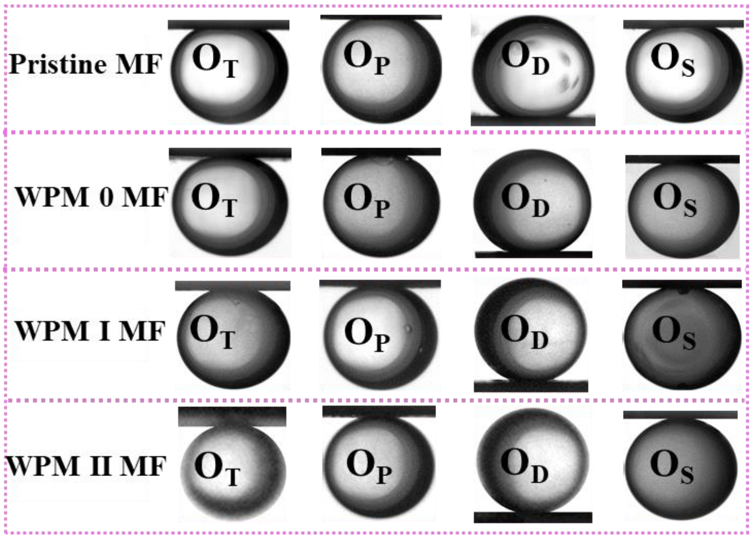

Supplementary Figure 16| Photographs of the underwater oil droplets on the surface of different membranes (O_T is UOCA of toluene on the membrane surface; O_P is UOCA of petroleum ether on the membrane surface; O_D is UOCA of dichloromethane on the membrane surface; O_S is UOCA of silicone oil on the membrane surface).

Compared with the original MF, under water oleophobic performance of the PCA/AP0.4 membrane was enhanced (UOCA were about 140 °). Furthermore, both of PCA/AP0.8 and PCA/AP1.6 MF had underwater oleophobicity with UOCA all greater than 145 °. In addition, after modified by Fe^{3+}, WPM MFs possessed stronger underwater superoleophobicity properties than that of the corresponding PCA/AP MFs (see Supplementary Figs. 14-16). The result was mainly attributed to the synergistic effect of the construction of micro/nano structure and abundant hydrophilic groups.

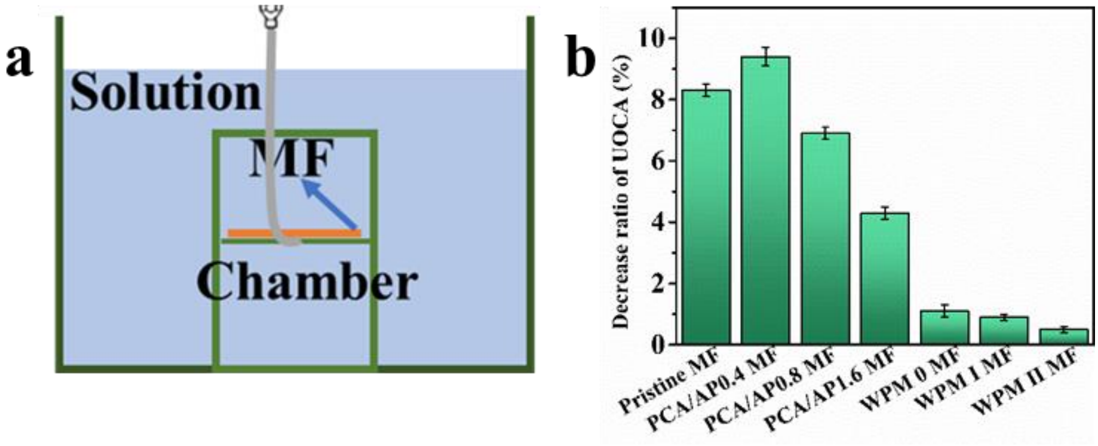

Supplementary Figure 17| (a) Device suitable for testing UOCA and **(b)** The decrease ratio of the UOCA values for each membrane after immersed in alkaline aqueous solution (pH=13) for 24 h. The error bar represents the standard deviation of the measurements.

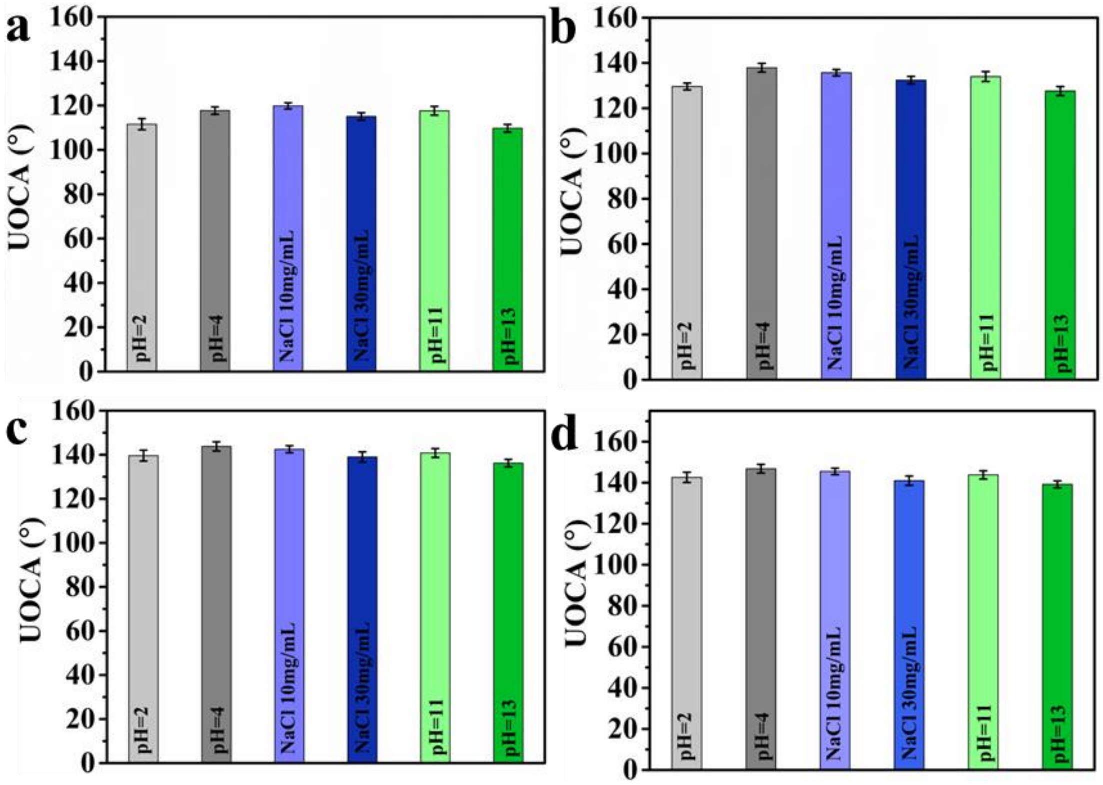

Supplementary Figure 18| (a) The pristine MF; **(b)** PCA/AP0.4; **(c)** PCA/AP0.8; **(d)** PCA/AP1.6. The error bar represents the standard deviation of the measurements.

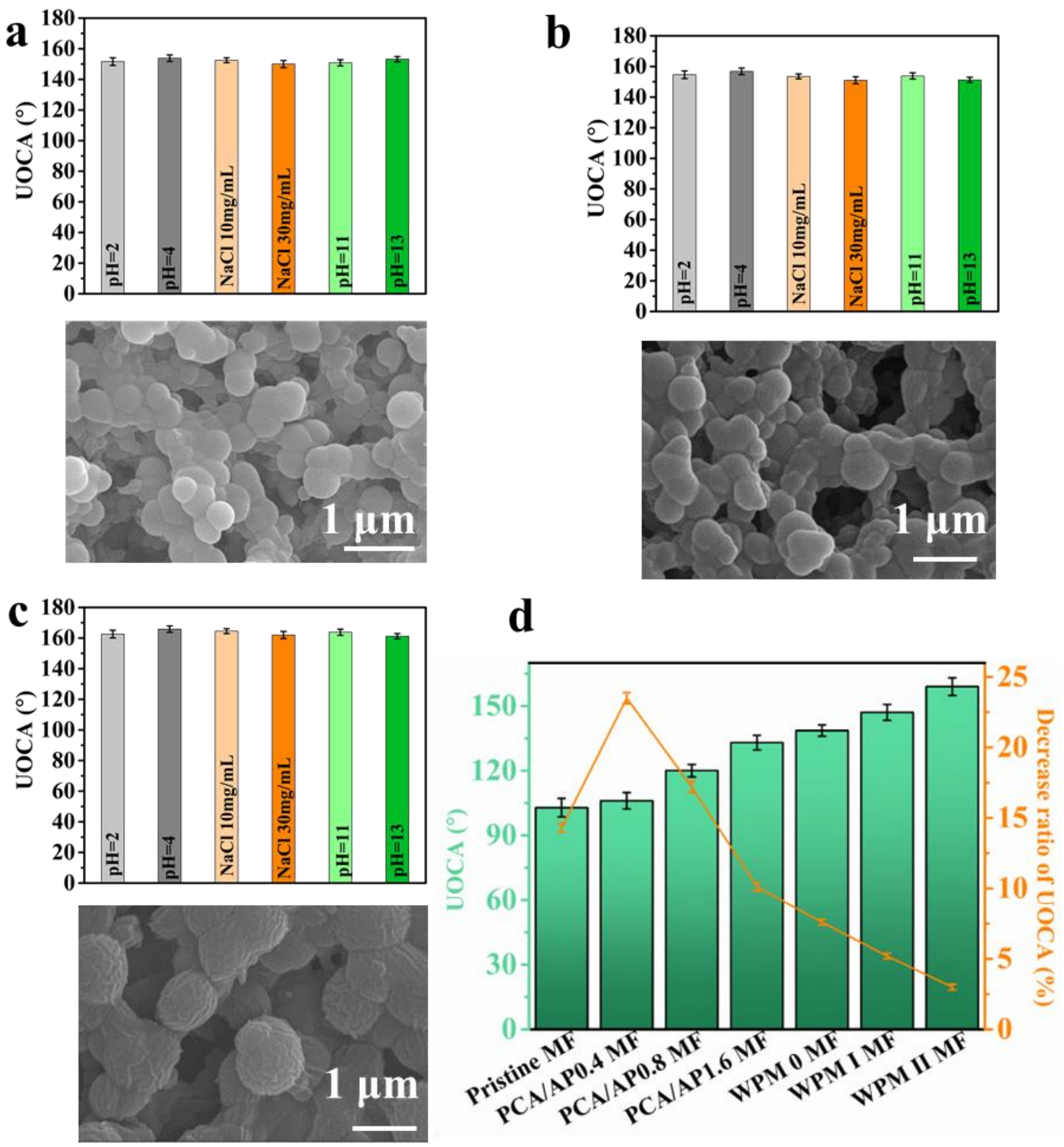

Supplementary Figure 19| The UOCA of **(a)** WPM 0; **(b)** WPM I; **(c)** WPM II MF after immersed in acidic, alkaline aqueous solution or saline solution for 24 h. **(d)** UOCA and the decrease ratio of the values for each membrane after immersed in acidic solution for 7 days. The error bar represents the standard deviation of the measurements.

To futher invesitgate the chemical stability of all membranes, we detected the UOCA of the membranes after immersing it into acidic, alkaline or saline solution for 24 h (see Supplementary Figs. 17-19). The results show the well chemical stability of WPM MFs, while UOCAs of PCA/AP membranes showed a downward trend under the acid, base or saline solution environment. Because of the greater drop of UOCA after immersed in alkaline aqueous solution (pH=13) for 24 h, we further tested the decrease ratio of

the UOCA values for each membrane after immersed in alkaline aqueous solution (pH=13) for 24 h. The results showed that the decrease ratio of UOCA of PCA/AP MFs decreased with the increase of APTES content, indicating that the increase of hydrophilic microparticles coating was conducive to the chemical stability of the membrane. In addition, we tested the UOCA and the decrease ratio of UOCA of each membrane after immersed in alkaline aqueous solution (pH=13) for 7 days to give the more powerful proof of the more stable underwater high oleophobic properties of WPM MFs. The results indicated that when the soaking time increased, the values of the original and PCA/AP MFs would decrease more and the decrease ratio was larger, showing they cannot be used in long-term oil/water separation. While, WPM MFs still retained high underwater oleophobic properties, especially UOCA of WPM II MF maintained above 160 ° and possessed stable underwater superoleophobicity property.

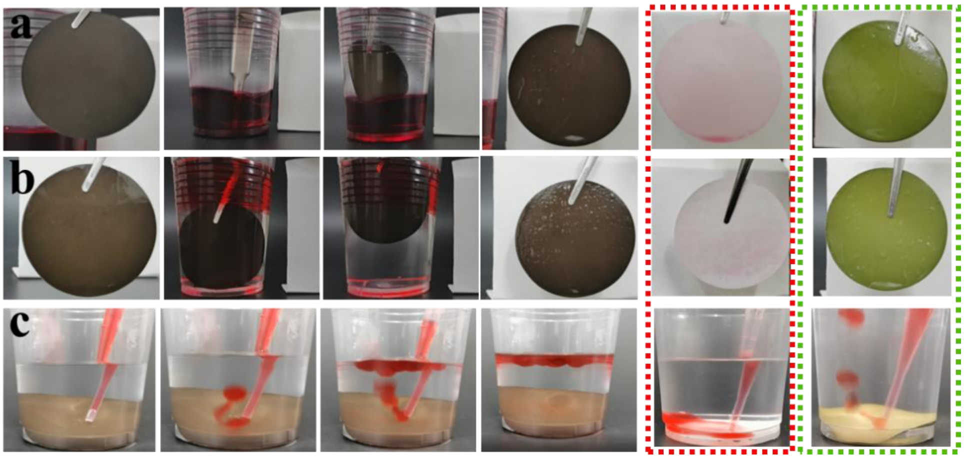

Supplemental Figure 20| Anti-low viscous oil fouling performance of the pristine, PCA/AP1.6 and WPM II MF. **(a)** The heavy oil fouling resistance; **(b, c)** The light oil fouling resistance. All oils are dyed red.

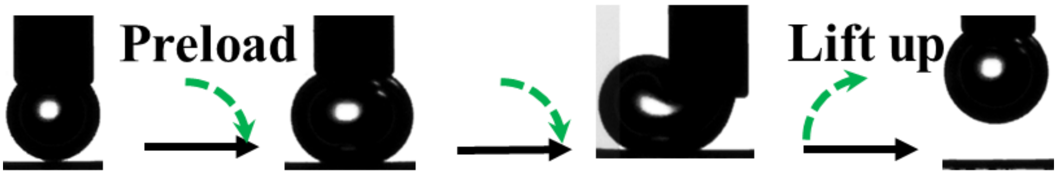

Supplemental Figure 21| Underwater oil adhesion process on WPM II MF.

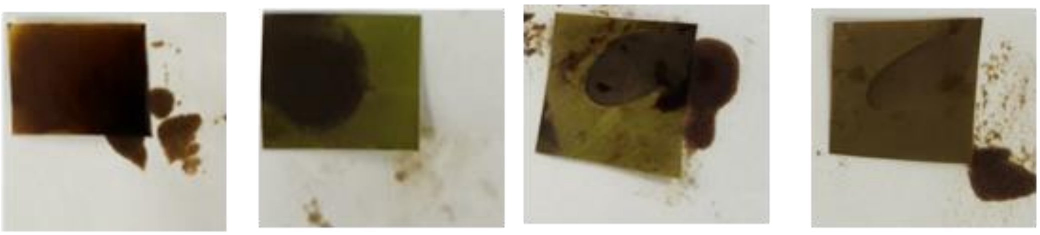

Supplemental Figure 22| Anti- dilute crude oil fouling performance of the membranes.

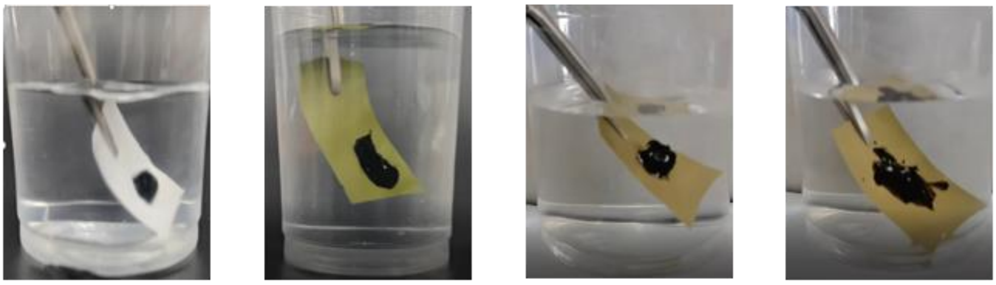

Supplemental Figure 23| Anti- dilute crude oil fouling performance of the membranes.

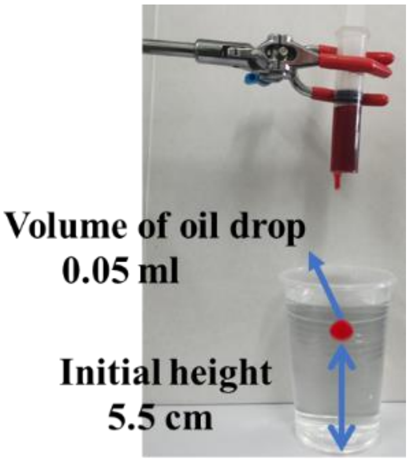

Supplemental Figure 24| Schematic of Free-falling experiment.

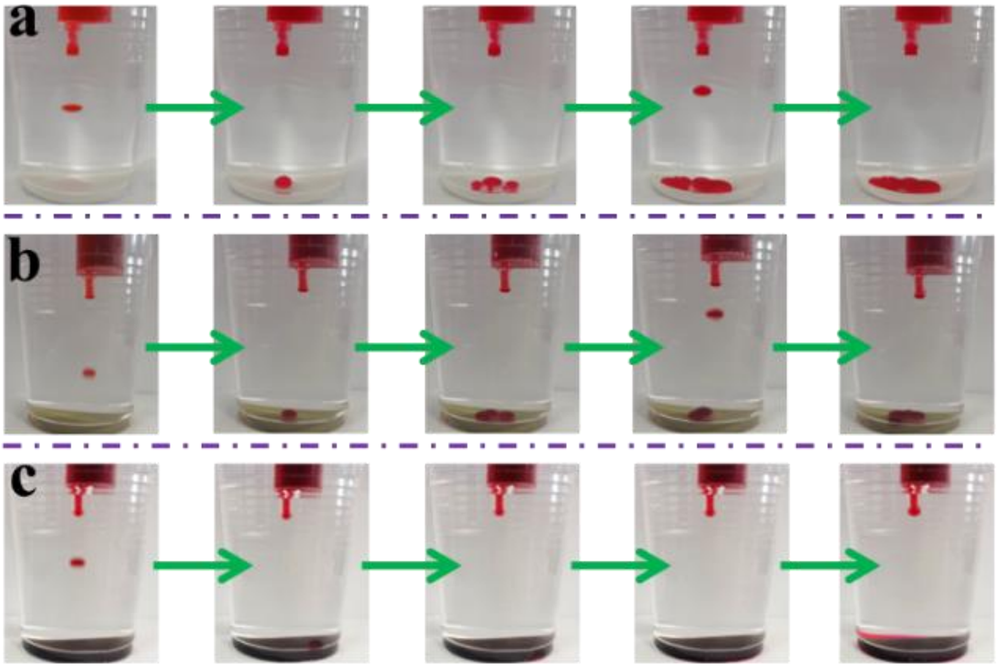

Supplemental Figure 25| Free-falling experiment of oil droplets in water on **(a)** The pristine; **(b)** WPM 0; **(c)** WPM II MF.

We tested the free-falling experiment for each membrane. After oil droplet fell on the surface of the pristine MF, it firmly adhered to the placement, and not fell off even if shaken violently. The oil droplets rolled from the falling point to other positions of the surface of PCA/AP MFs, WPM 0 MF and WPM I MF finally stop at a certain position on the membrane surface. However, oil drops immediately bounced up like a ball after touched WPM II MF surface, then slipped into the water after touching the membrane surface again. The results illustrated different from other non-migratory foulant, oil fouling not only contaminated the impact point, but migrated or amalgamated at other positions on the membrane surface, thus improving the resistance to oil fouling was more challenging. Also, the different trajectories of oil droplets contacting the surfaces of WPM 0 and WPM II MF indicated that their anti-oil-fouling mechanisms were different.

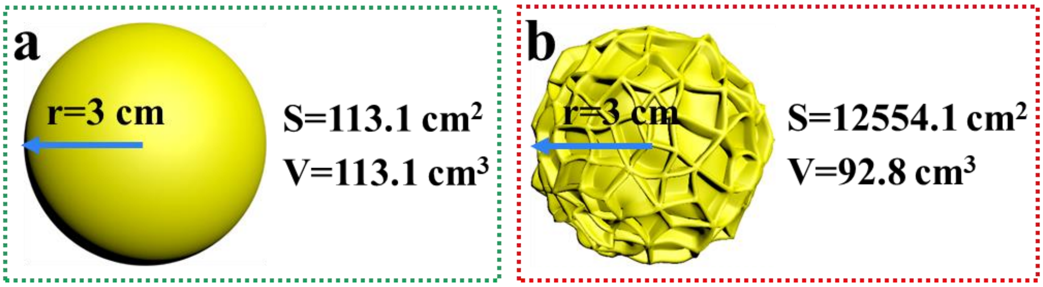

Supplemental Figure 26| The surface area and volume of the smooth microparticles (**a**, PCA/AP1.6) and the corresponding microparticles with wrinkled patterns (**b**, WPM II) were calculated by simulation.

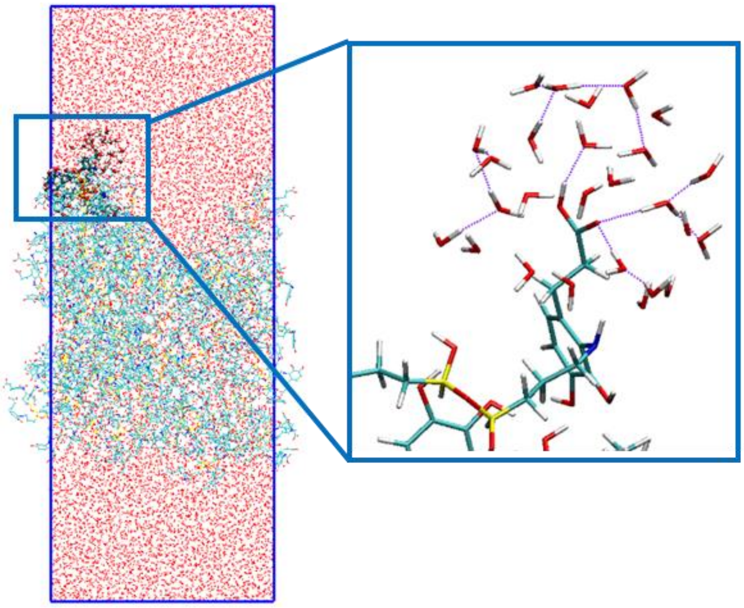

Supplemental Figure 27| Structure of PCA/AP1.6 MF/water molecular with enhanced hydrogen bonds formed between membranes and hydration layer water.

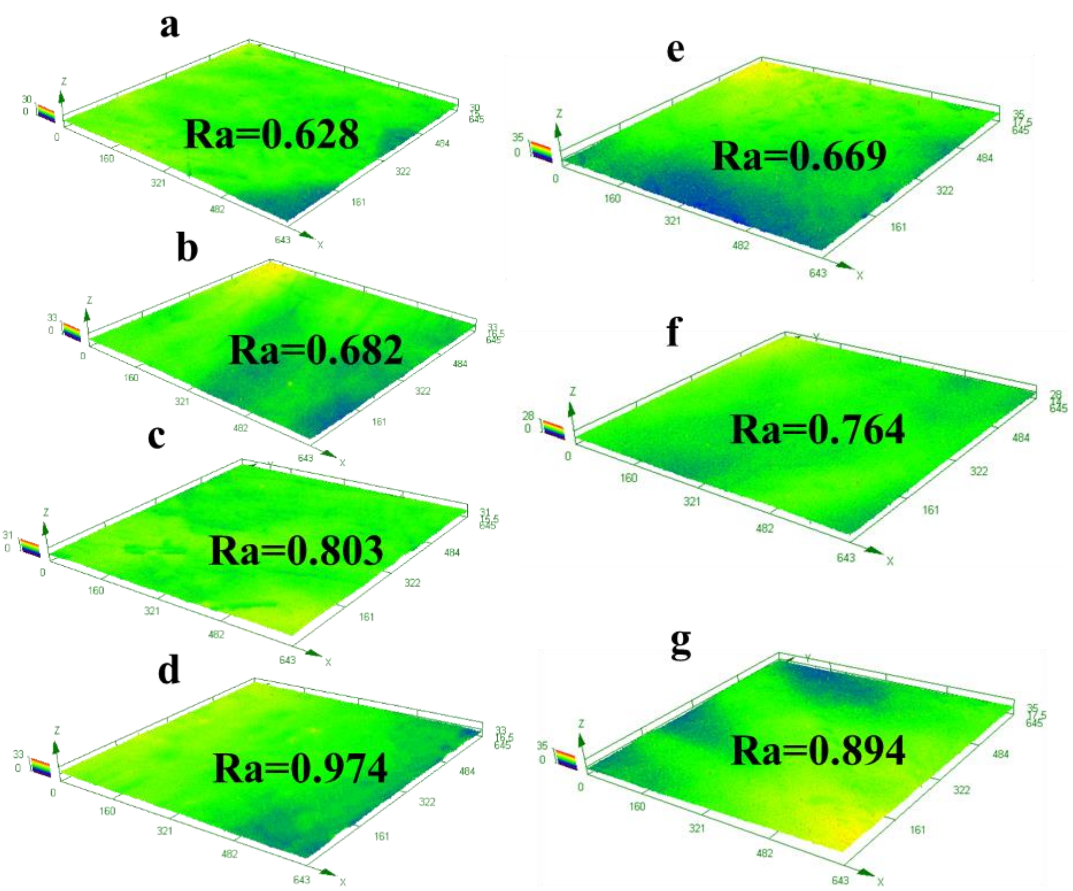

Supplementary Figure 28| CLSM images of **(a)** the pristine; **(b)** PCA/AP0.4; **(c)** PCA/AP0.8; **(d)** PCA/AP1.6; **(e)** WPM 0; **(f)** WPM I; **(g)** WPM II MF.

The surface roughness of the WPM 0 MF (Ra=0.669 μm) increased by 6.6% compared to that of the pristine MF (Ra=0.628 μm) and decreased by 1.9% compared to that of the PCA/AP0.4 MF (Ra=0.682 μm). The surface roughness of WPM I MF (Ra=0.764 μm) increased 25% than that of the pristine MF and decreased 4.9% than that of PCA/AP0.8 MF (Ra=0.803 μm), respectively. Besides, the roughness of WPM II MF (Ra=0.894 μm) was 8.2% lower than that of PCA/AP1.6 MF (Ra=0.974 μm) and 42% higher than that of the pristine MF. The largest size of PCA/AP1.6 microparticles caused the average distance between the contour peak line and the membrane surface became higher after coating on the membrane surface, resulting in the maximum roughness. While, the wrinkling formed by WPM microparticles would reduce the average distance between the microparticles surface contour and the membrane surface and then the roughness of WPM MFs declined.

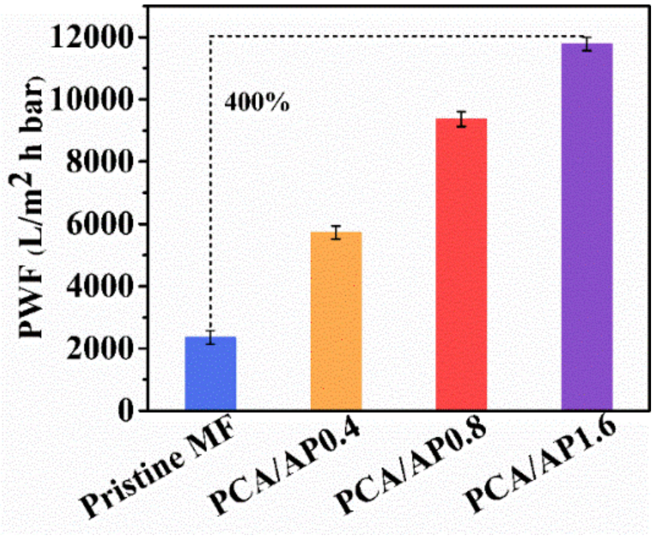

Supplementary Figure 29| The pure water flux of different membranes.

The PWF of WPM I MF reached 12522 L m^{-2} h^{-1} bar, a 510% and 350% improvement when compared to commercial pristine and PCA/AP0.8 MF. The PWF of WPM 0 MF reached 7800 L m^{-2} h^{-1} bar, a 290% and 270% improvement when compared to commercial pristine and PCA/AP0.4 MF (see Supplementary Fig. 29).

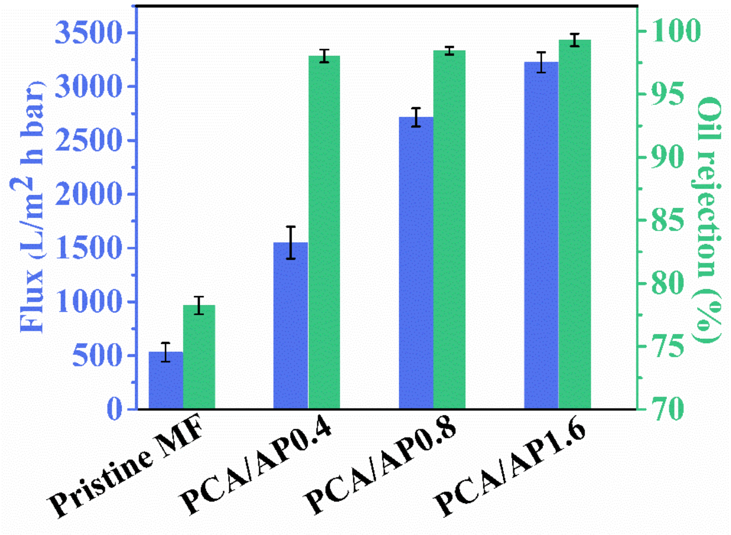

Supplementary Figure 30| The filtration flux and oil rejection of different membranes.

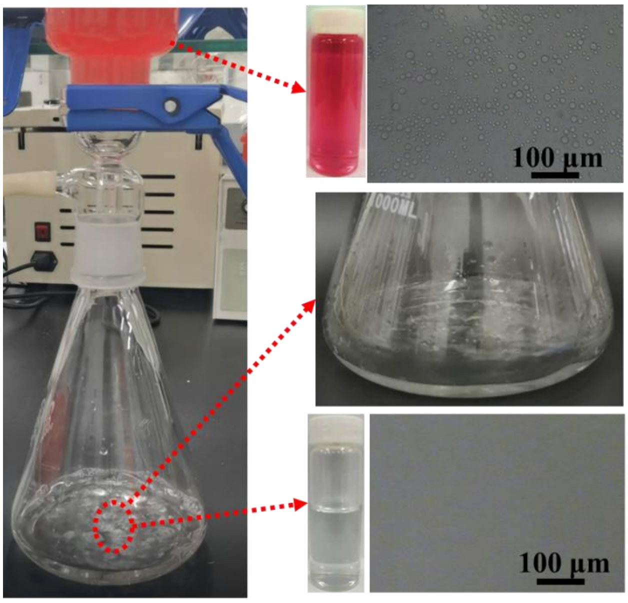

Supplementary Figure 31| The photographs of filtration process and optical microscope images of T/W emulsion before and after filtration

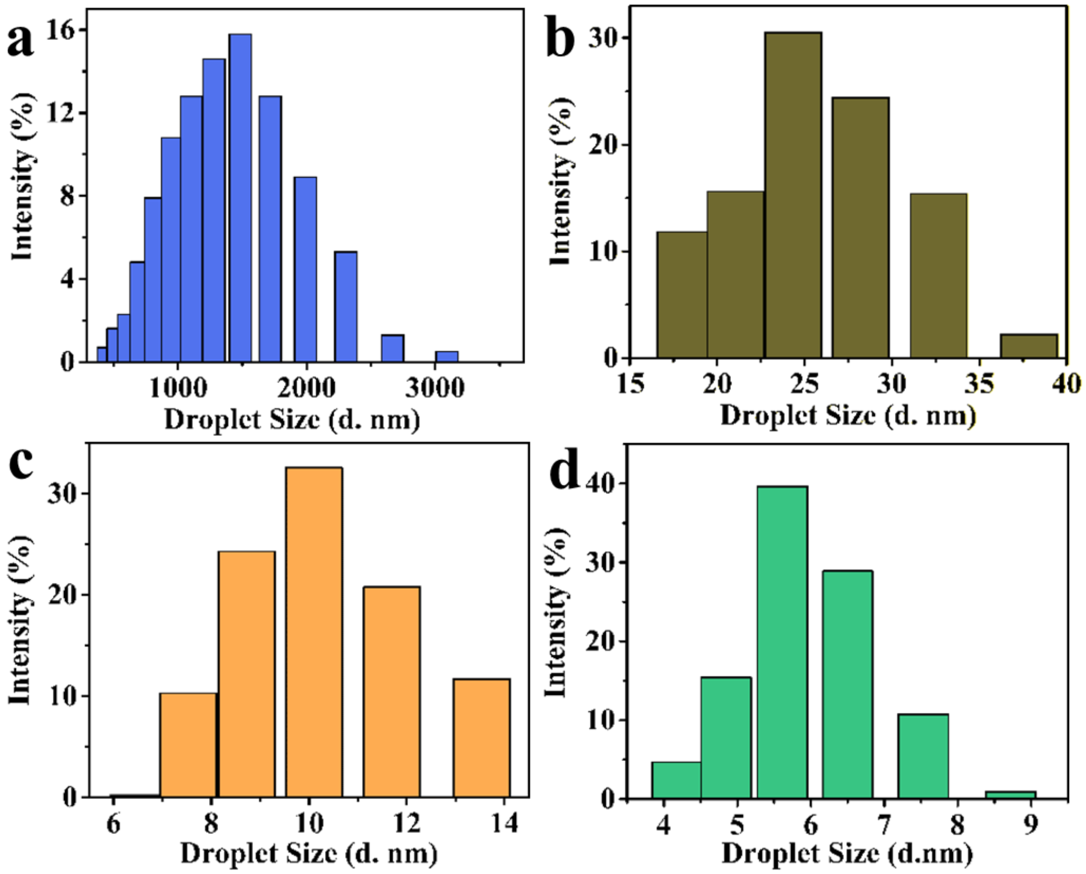

Supplementary Figure 32| (a) The oil drops in the feed of T/W emulsion before separation; **(b-d)** The oil drops in the filtration after T/W emulsion separation of WPM 0, WPM I, WPM II MF.

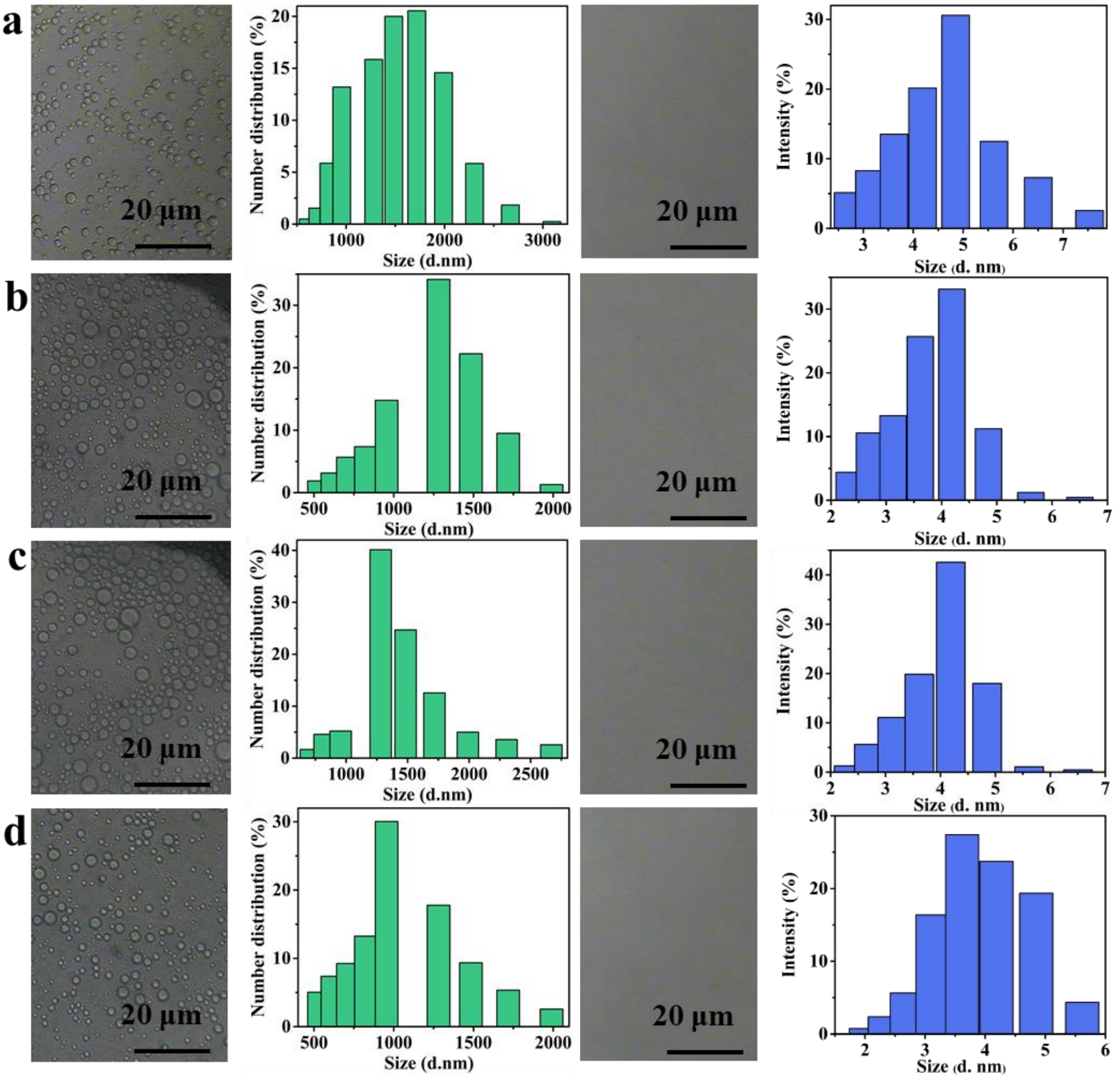

Supplementary Figure 33| The microscopic images and the oil drops in the feed/ filtration of /W emulsion of WPM II MF. **(a)** D/W; **(b)** P/W; **(c)** Si/W; **(d)** H/W.

The O/W emulsion before filtration was deep pink with a particle size of about 1500 nm, by contrast, the filtrate was almost transparent with no droplets, which was further confirmed by optical microscopy (see Supplementary Figs. 31-33). After WPM 0 and WPM I membrane filtration, more than 10 nm oil droplets still penetrated the membrane, but no 10 nm oil droplets permeated across the WPM II MF, indicating that the WPM II MF had the optimal O/W separation performance. Then we tested the separation performance of WPM II MF for different O/W emulsions, and it can be seen that the particle size of the emulsion in the filtrate filtered by the membrane can reach less than 5 nm.

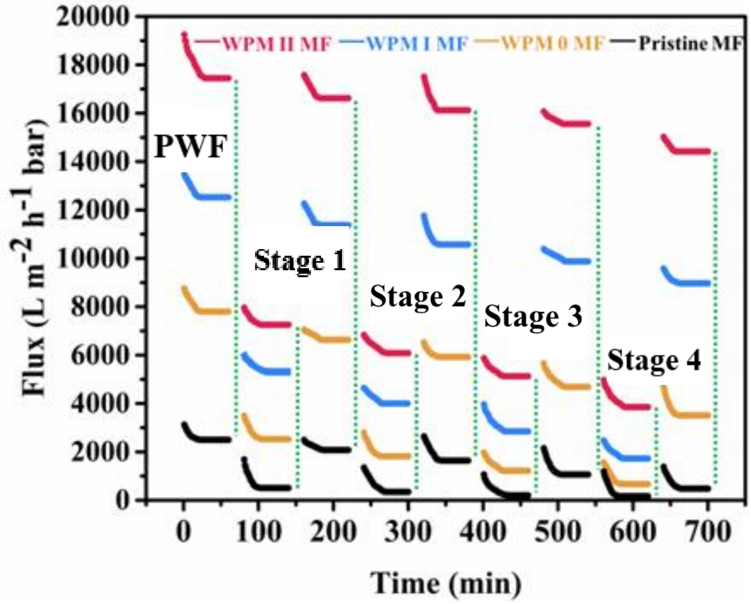

Supplementary Figure 34| The separation performance of membranes during four-cycles emulsion separation.

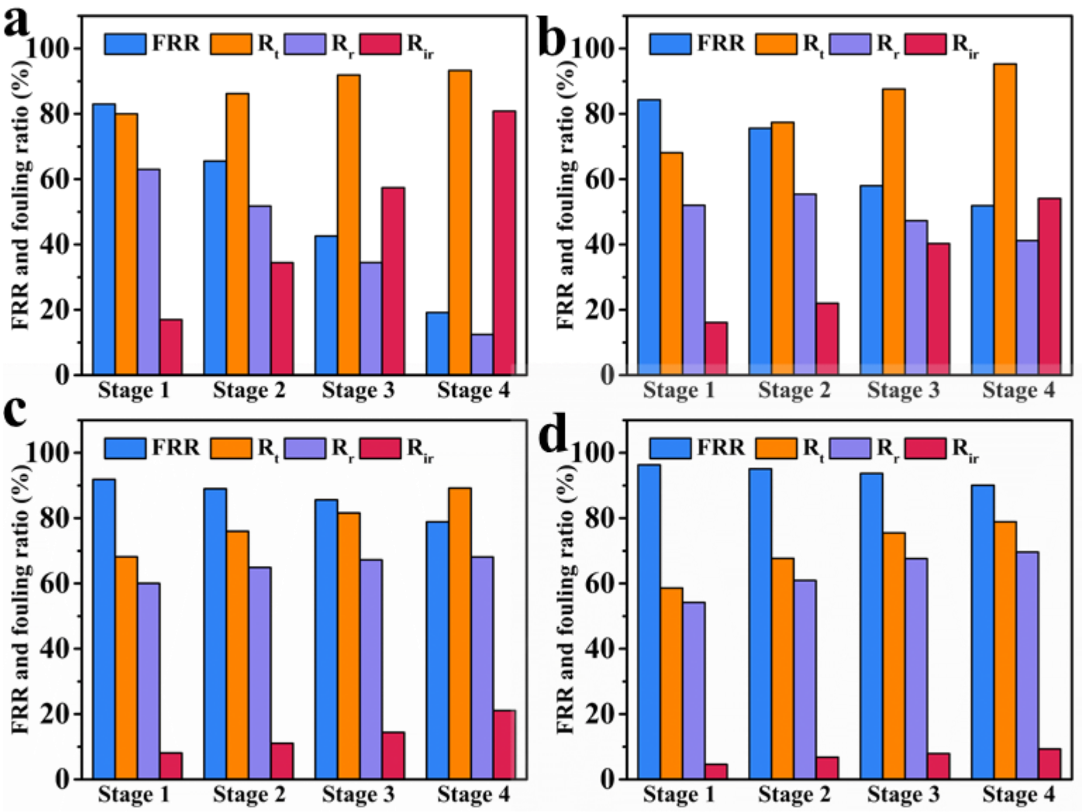

Supplementary Figure 35| FRR, R_t R_r, R_{ir} values of **(a)** Pristine; **(b)** WPM 0; **(c)** WPM I; **(d)** WPM II MF during four-cycles emulsion separation.

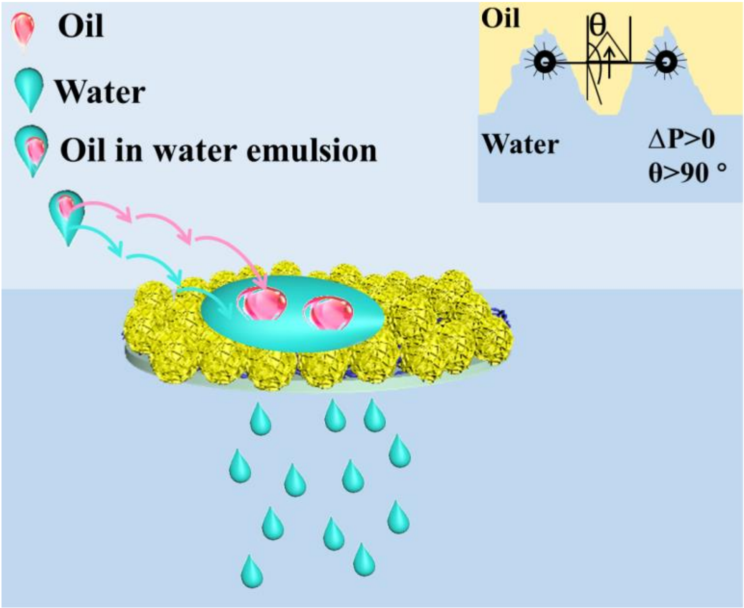

Supplementary Figure 36| Schematic of demulsification mechanism of WPM II MF for crude-oil/water separation.

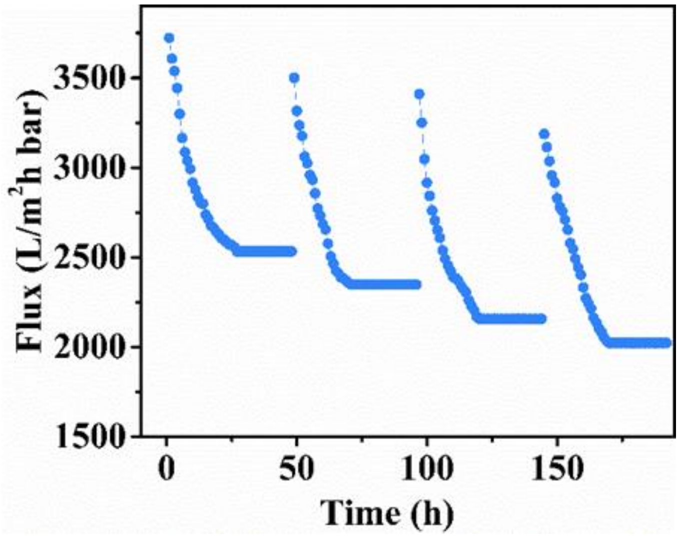

Supplementary Figure 37| Separation performance of viscous crude oil/water mixture of WPM II MF under 1 bar.

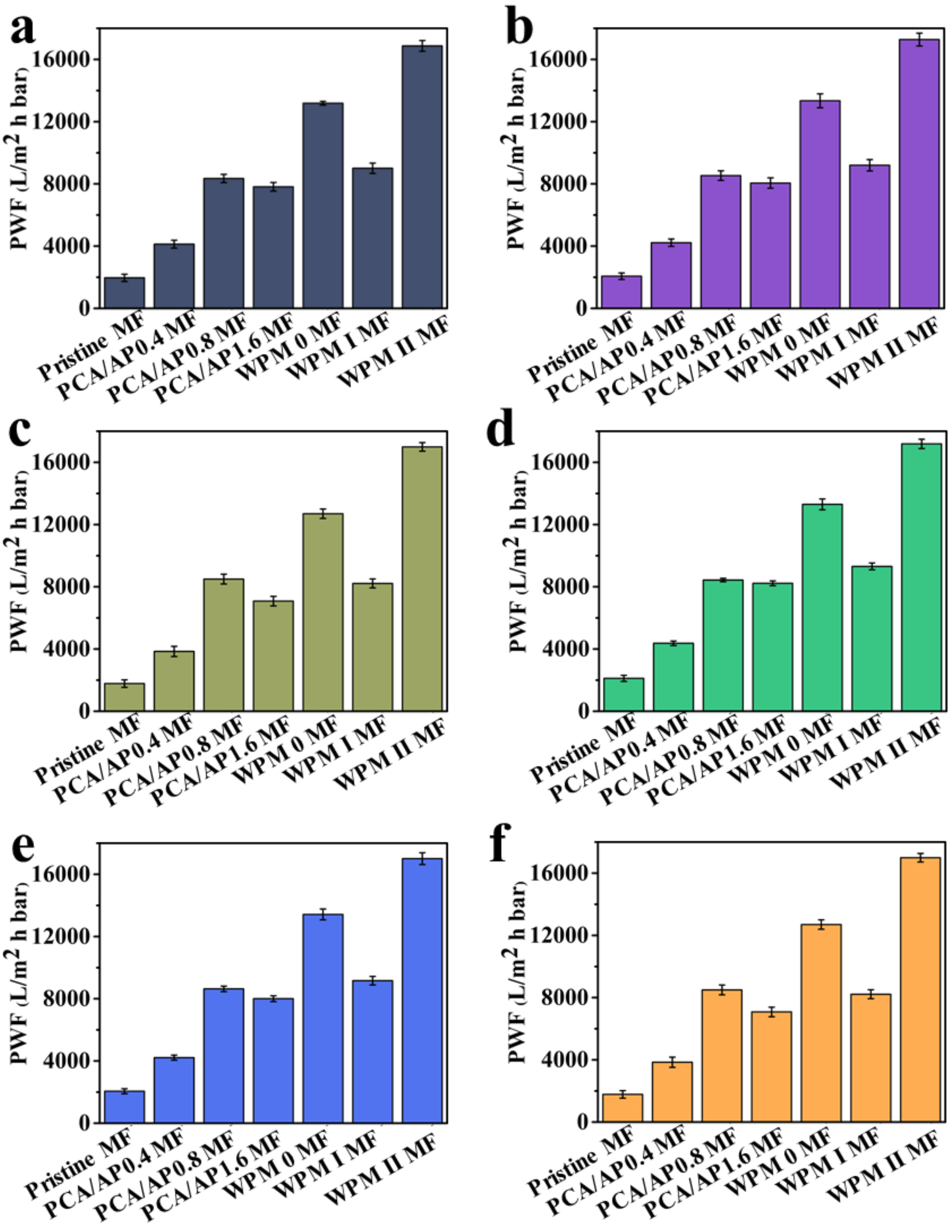

Supplementary Figure 38| The pure water flux of different membranes after immersed in acidic, alkaline aqueous solution or saline solution for 24 h. **(a)** pH=2; **(b)** pH=4; **(c)** NaCl 10 mg/ml; **(d)** NaCl 30 mg/ml; **(e)** pH=11; **(f)** pH=13.

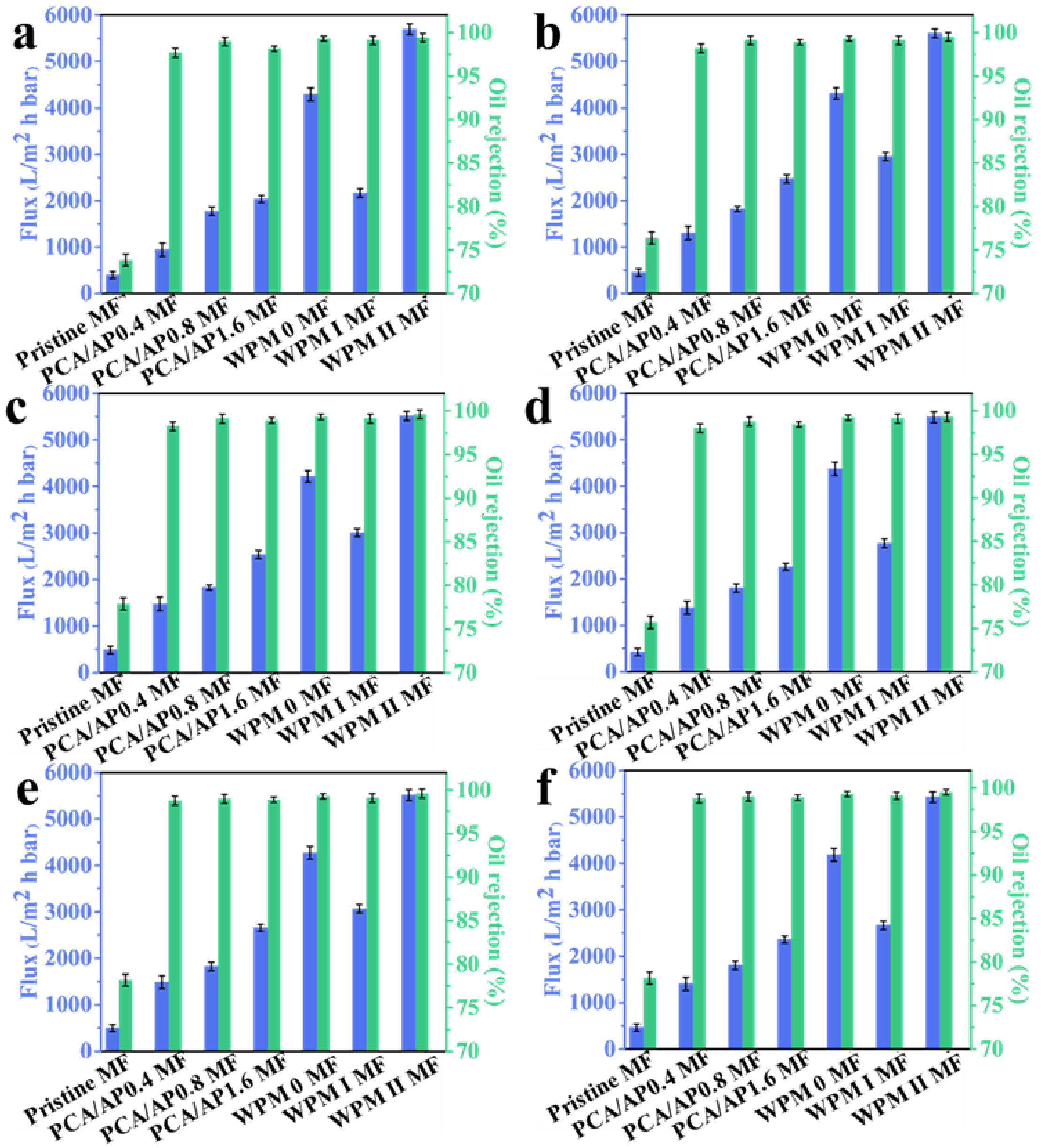

Supplementary Figure 39| The filtration flux and oil rejection of different membranes after immersed in acidic, alkaline aqueous solution or saline solution for 24 h. **(a)** pH=2; **(b)** pH=4; **(c)**NaCl 10 mg/ml; **(d)** NaCl 30 mg/ml; **(e)** pH=11; **(f)** pH=13.

Afterwards, we examined the stability of the pure water flux and the separation performance of all the membranes under the acid, base or saline solution environment, and the results illustrated that the separation performance of membranes modified by only APTES decreased sharply, and membranes after introduction Fe ions still had high flux and precise separation performance in acid, base or salt environments.

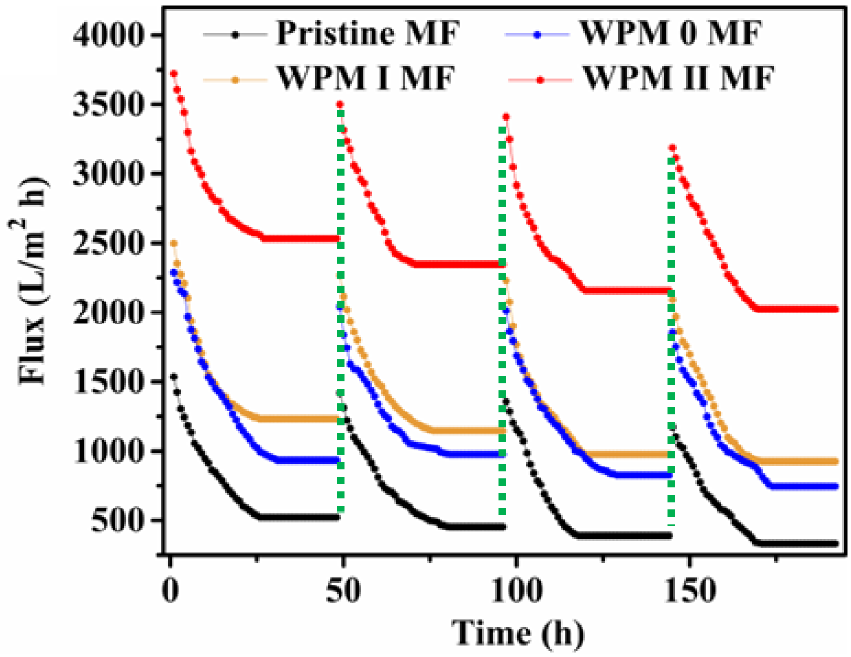

Supplementary Figure 40| Long-term water flux of WPM II MF.

Maintaining the long-term separation performance of the membrane is a key factor in determining its practical application. Thus, we tested the flux of WPM II MF to evaluate long-term operation stability within 190 h for C/W emulsion separation under 1 bar of cross-flow filtration. The flux of WPM II MF in Supplementary Fig. 40 showed the separation flux still maintained above 2000 L m^{-2} h^{-1} bar after 190 hours of operation, which had a well long-term operation stability under cross-flow filtration mode, illustrating WPM II MF has potential application in oily wastewater treatment.

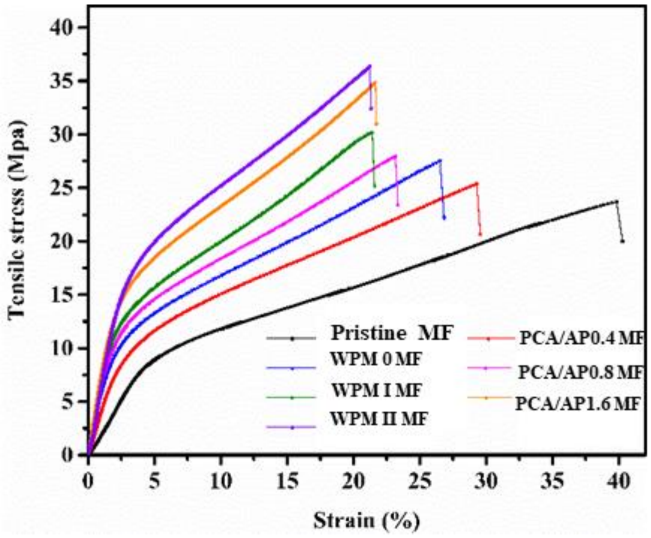

Supplementary Figure 41| Mechanical properties using tensile tests of membranes.

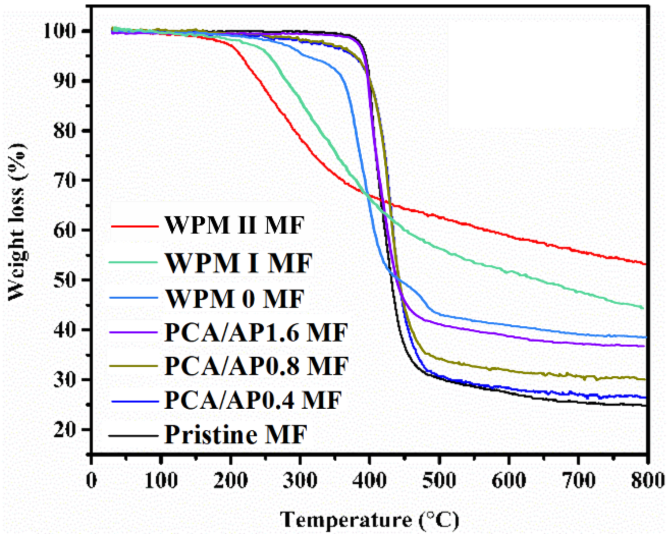

Supplementary Figure 42| TGA of membranes.

Supplementary Table 1| The detailed modification conditions and abbreviations of the membranes.

Membranes	CA (g)	APTES (g)	$FeCl_3$ (mg/ml)	Reaction Time (h)	Temperature (°C)
Pristine MF	0	0	0	12	25
PCA/AP0.4 MF	0.2	0.4	0	12	25
WPM 0 MF	0.2	0.4	100	12	25
PCA/AP0.8 MF	0.2	0.8	0	12	25
WPM I MF	0.2	0.8	100	12	25
PCA/AP1.6 MF	0.2	1.6	0	12	25
WPM II MF	0.2	1.6	100	12	25

Supplementary Table 2| Summary of the DFT simulation results.

System	Binding energy (kJ/mol)	$E_{complex}$ (kJ/mol)	$E_{fragment1}$ (kJ/mol)	$E_{fragment2}$ (kJ/mol)
Fe^{3+}-OH	-151.79	-3049.642	-1786.377	-1263.208

Fe^{3+}-NH	-221.93	-3049.663	-1786.371	-1263.208
Fe^{3+}-COOH	-217.48	-3049.666	-1786.375	- 1263.208

Supplementary Table 3| Surface elemental analysis of the membranes from the XPS spectra.

Samples	Elemental and Area/%					
	C *1s*	N *1s*	F *1s*	O *1s*	Si *2p*	Fe *2p3*
Pristine MF	59.11	1.77	34.72	4.05	0.35	0
PCA/AP0.4 MF	60.58	5.28	1.39	23.06	9.7	0
WPM 0 MF	63.19	4.4	2.18	22.4	7.42	0.41
PCA/AP0.8 MF	57.78	6.91	1.53	22.75	11.02	0
WPM IMF	59.23	4.29	2.17	25.55	6.18	2.58
PCA/AP1.6 MF	57.23	6.85	2.94	21.34	11.65	0
WPM IIMF	55.24	3.74	1.23	30.38	5.64	3.78

Supplementary Table 4| XPS results of all membranes.

Samples	Elemental and Atomic /%	POS.	Peak attribution	Area/%	Molar fraction/%
Pristine MF	C *1s* (59.11)	283.54	C-C	51.98	30.73
		285.46	C-O	45.11	26.66
		291.08	C-F	2.91	1.72
	O *1s* (4.05)	530.27	C-O	100	4.05
	N *1s* (1.77)	400.81	N-H	100	1.77
PCA/AP0.4 MF	C *1s* (60.58)	284.60	C-C/C=C	17.12	10.37
		287.88	C-O	14.91	9.03

		290.84	C-F	12.02	7.28
		283.86	C-Si	15.39	9.32
		287.88	C=O	13.58	8.23
		286.24	C=N	13.86	8.40
		285.76	C-N	13.13	7.95
	O *1s*	533.48	-OH	58.04	13.38
	(23.06)	530.67	-O-C	25.42	5.86
		529.38	O=C	16.54	3.81
	N *1s*	401.32	N-H	55.88	2.95
	(5.28)	399.38	N=C	44.12	2.33
WPM 0	C *1s*	291.07	C-F	0.12	0.08
MF	(63.19)	287.85	C=O	6.55	4.14
		286.21	C=N	22.44	14.18
		285.35	C-N	22.32	14.11
		284.81	C-O	17.93	11.33
		284.31	C=C/C-C	22.28	14.08
		283.84	C-Si	8.35	5.28
	O *1s*	533.36	-O-C	13.12	2.94
	(22.4)	532.69	O=C	34.80	7.8
		531.97	-OH	17.16	3.84
		531.06	O-Fe	34.92	7.82
	N *1s* (4.4)	401.61	N-H	50.25	2.21
		399.50	N=C	39.74	1.75
		395.61	N-Fe	10.01	0.44
PCA/AP0.8	C *1s*	291.36	C-F	2.33	1.35
MF	(57.78)	287.76	C=O	8.08	4.67
		286.10	C=N	14.07	8.13
		285.26	C-N	10.47	6.05

		284.83	C-O	17.95	10.37
		284.22	C C/C=C	19.66	11.36
		283.76	C-Si	27.44	15.85
	O *1s*	532.72	-O-C	16.84	3.83
	(22.75)	531.93	O=C	41.02	9.33
		531.11	-OH	42.15	9.59
	N *1s*	401.32	N-H	46.45	3.21
	(6.91)	399.38	N=C	53.55	3.70
WPM I MF	C *1s*	291.07	C-F	0.12	0.08
	(59.23)	287.85	C=O	6.55	4.14
		286.21	C=N	22.44	14.18
		285.35	C-N	22.32	14.11
		284.81	C-O	17.93	11.33
		284.31	C=C/C-C	22.28	14.08
		283.84	C-Si	8.35	5.28
	O *1s*	529.70	O-Fe	22.95	5.86
	(25.55)	530.97	-OH	36.66	9.37
		531.99	O=C	26.34	6.73
		533.05	-O-C	14.04	3.59
	N *1s*	401.22	N-H	48.63	2.08
	(4.29)	399.00	N=C	34.20	1.47
		399.86	N-Fe	17.18	0.74
PCA/AP1.6 MF	C *1s*	290.85	C-F	4.80	2.74
	(57.23)	287.96	C=O	11.24	6.43
		286.46	C=N	17.60	10.07
		285.66	C-N	18.18	10.41
		285.00	C-O	20.73	11.86
		284.50	C=C/C-C	24.07	13.78
		284.02	C-Si	3.38	1.93

	O *1s*	532.59	-O-C	24.33	5.19
	(21.34)	531.93	O=C	58.30	12.44
		530.74	-OH	17.37	3.71
	N *1s*	400.95	N-H	38.77	2.66
	(6.85)	399.12	N=C	61.23	4.19
WPM II	C *1s*	291.08	C-F	0.13	0.07
MF	(55.24)	287.73	C=O	5.91	3.26
		286.14	C=N	22.13	12.22
		285.25	C-N	22.01	12.16
		284.71	C-O	18.39	10.16
		284.23	C=C/C-C	22.86	12.63
		283.79	C-Si	8.57	4.73
	O *1s*	529.33	O-Fe	18.24	5.54
	(30.38)	530.61	-OH	33.81	10.27
		531.71	O=C	31.39	9.54
		533.23	-O-C	16.55	5.03
	N *1s*	401.21	N-H	33.47	1.25
	(3.74)	398.52	N=C	34.86	1.30
		399.58	N-Fe	31.67	1.19

Supplementary Table 5| The viscosity of various liquids.

Samples	Viscosity (mPa·s)	Temperature (℃)
Petroleum ether	0.3	20
Dichloroethane	0.8	20
Dilute crude oil	44	20
Viscous crude oil	9630	20

Supplementary Table 6|. Summary of the MD simulation of PCA/AP1.6 and WPM II MF, including binding and dissociation energies, diffusion coefficients of hydration layer water.

System	ΔE^{+} (kJ/mol)	ΔE^{-} (kJ/mol)	Diffusion coefficients ($m^{2/s}$)	Residence Time (ps)
PCA/AP1.6 MF	0.4	2.04	3.84±0.07	4.5
WPM II MF	0.5	3.0	1.95±0.17	8.22

Supplementary Table 7| Polar and disperse components of the liquids.

Liquids	Surface-energy components (mN/m)		
	γ_l	γ_l^d	γ_l^p
Water	72.8	21.8	51.0
Ethylene glycol	48.0	29.0	19.0

Supplementary Table 8|Surface energy of PCA/AP1.6 and WPM II MF.

Sample membranes	Contact angle (°)		Surface-energy components (mN/m)		
	Water	Ethylene glycol	γ_s	γ_s^d	γ_s^p
PCA/AP1.6 MF	6	10	84.3	2.5	81.8
WPM II MF	0	18	88.2	1.5	86.8

Supplementary Table 9| O/W emulsion separation permeance information of WPM II MF and high-performance membranes reported in open literatures[2-28].

Membrane	Modification strategy	Model oil	Surfactant	Pure water flux	Permeability (L·m−2	Oil rejection (%)	Trans-membrane	Reference

					h−1)		pressure (bar)	
PVDF/TA-PEI/Ti^{4+}	Coating	Pump oil	SDS	6736	3139	99.9	0.5	2
PVDF-PG/PEI	Coating	Toluene	SDS	1333	1053	98.4	0.1	3
PA@PEI/PVDF	Coating	Toluene	Tween 80	/	1343	99.1	0.15	4
Janus PVDF	Pray-coating	Toluene	Tween 80	/	2060	94	1	5
HNTs@PVDF@PDA/PSBMA	Coating	Soybean oi	SDS	/	2365	99.8	0.85	6
UF-T/C	Coating	Toluene	Tween 80	7900	2000	99.5	0.9	7
CNT@PDA-ZT	Nano-assembly	n-Hexane	SDS	3360	1880	99.6	0.5	8
MF-DA/TA	Coating	Toluene	Tween 80	13500	2100	99.6	0.09	9
GO/LDH	LBL	Decane			1900	93	1	10
Cu^{2+}/alginate/PVDF	Coating	Crude oil	SDS	1600	1230	99.8	1	11
PP/PDA-PVP	Coating	SO	DC193	440	268	98	1	12
PVDF/CS&DA	Coating	PO	SDS	201	171	99.7	0.1	13
GA-APTES/PV	Coating	Toluene	Tween 80	10273	1000	99.5	0.9	14

DF								
PK-g-PSBMA	Coating	Toluene	SDS	1820	1710	99.7	1	15
SWCNT/PD/PEI	Nano-assembly	Industrial oil	Tween 80	7270	6060	99.99	0.5	16
Pal coated membrane	Coating	kerosene	Tween 80	/	477.7	99.6	0.8	17
PVDF-TA-SP	Coating	Glycerol	SDS	2250	1800	99	0.6	18
loess-coated PVDF	Coating	Petroleum ether	Tween 80	7000	910.4	99.6	1	19
PVDF@pDA@SiO_2	Coating	Dichloromethane	Tween 80	/	572	98	0.8	20
MF-T/K	Coating	Toluene	Tween 80	/	2580	99.2	1	21
PVDF-TAAP	Coating	Toluene	Tween 80	6364	2500	99.6	1	22
TA-APTES-TEOS	Coating	Toluene	Tween 20	10384	2650	99.7	1	23
TA-APTES-Fe	Coating	Toluene	SDS	/	2200	99	1	24
PVDF-POSS-NH_2/FPN	Coating	Toluene	Tween 80	13000	2700	99.5	1	25
MF-C/A	Coating	Toluene	Tween 80	13000	1300	99	1	26
Coating-	Coating	Toluene	/	/	2750	99	1	27

deposited copper mesh								
chitin NFs	Vacuum-filtering	Toluene	Tween 80	2630	563	91	0.95	28
WPM II	Coating	Petroleum ether	Tween 80	17555.5	5635	99.6	1	This work

Supplementary Table 10| Mechanical properties of different membranes.

Ionomer	E/MPa	TS/MPa	ε/%
Pristine MF	1.53	23.72	39.87
PCA/AP0.4 MF	3.17	25.5	29.29
WPM 0 MF	5.63	27.52	26.57
PCA/AP0.8 MF	5.92	27.95	23.13
WPM I MF	6.81	30.25	21.5
PCA/AP1.6 MF	6.66	34.93	21.72
WPM II MF	7.69	36.47	21.31

Reference

1. Ohno, S., Hori, W., Hosokawa, M., Tatsuzawa, F. & Doi, M. Post-transcriptional silencing of chalcone synthase is involved in phenotypic lability in petals and leaves of bicolor dahlia (Dahlia variabilis) 'Yuino'. *Planta* **247**, 413-428 (2018).

2. Zhao, X. *et al.* Engineering superwetting membranes through polyphenol-polycation-metal complexation for high-efficient oil/water separation: From polyphenol to tailored nanostructures. *J. Membr. Sci.* **630**, 119310 (2021).

3. Zuo, C. *et al.* Co-deposition of pyrogallol/polyethyleneimine on polymer membranes for highly efficient treatment of oil-in-water emulsion. *Sep. Pur. Tech.* **267**, 118660 (2021).

4. Zeng, X. *et al.* Fabrication of superhydrophilic PVDF membranes by one-step modification with eco-friendly phytic acid and polyethyleneimine complex for oil-in-water emulsions separation. *Chemosphere*

264, 128395 (2021).

5. Zuo, J.-H. *et al.* Janus polyvinylidene fluoride membranes fabricated with thermally induced phase separation and spray-coating technique for the separations of both W/O and O/W emulsions. *J. Membr. Sci.* **595**, 117475 (2020).

6. Zhou, Y. *et al.* A modified TA-APTES coating: Endowing porous membranes with uniform, durable superhydrophilicity and outstanding anti-crude oil-adhesion property via one-step process. *J. Membr. Sci.* **618**, 118703 (2021).

7. Yang, X., Yan, L. Ma, J. Bai, Y. & Shao, L. Bioadhesion-inspired surface engineering constructing robust, hydrophilic membranes for highly-efficient wastewater remediation. *J. Membr. Sci.* **591**, 117353 (2019).

8. Zhao, X. *et al.* Bioinspired synthesis of polyzwitterion/titania functionalized carbon nanotube membrane with superwetting property for efficient oil-in-water emulsion separation. *J. Membr. Sci.* **589**, 117257 (2019).

9. Yang, X., Yan, L., Wu, Y., Liu, Y. & Shao, L. Biomimetic hydrophilization engineering on membrane surface for highly-efficient water purification. *J. Membr. Sci.* **589**, 117223 (2019).

10. Zhao, X., Jia, N., Cheng, L., Liu, L. & Gao, C. Metal-polyphenol coordination networks: Towards engineering of antifouling hybrid membranes via in situ assembly. *J. Membr. Sci.* **563**, 435-446 (2018).

11. Jiang, J. *et al.* Antifouling and antimicrobial polymer membranes based on bioinspired polydopamine and strong hydrogen-bonded poly(n-vinyl pyrrolidone). *ACS Appl. Mater. Inter.* **5**, 12895-12904 (2013).

12. Zhang, G. *et al.* Bio-inspired underwater superoleophobic PVDF membranes for highly-efficient simultaneous removal of insoluble emulsified oils and soluble anionic dyes. *Chem. Eng. J.* **369**, 576-587 (2019).

13. Yang, X., Sun, H., Pal, A., Bai, Y. & Shao, L. Biomimetic silicification on membrane surface for highly efficient treatments of both oil-in-water emulsion and protein wastewater. *ACS Appl. Mater. Inter.* **10**, 29982-29991 (2018).

14. Gao, S. J., Zhu, Y. Z., Zhang, F. & Jin, J. Superwetting polymer-decorated SWCNT composite ultrathin films for ultrafast separation of oil-in-water nanoemulsions. *J. Mater. Chem. A* **3**, 2895-2902 (2015).

15. Zhang, L., Lin, Y., Cheng, L. Yang, Z. & Matsuyama, H. A comprehensively fouling- and solvent-resistant aliphatic polyketone membrane for high-flux filtration of difficult oil-in-water micro- and nanoemulsions. *J. Membr. Sci.* **582**, 48-58 (2019).

16. Cao, J. *et al.* Self-assembled MOF membranes with underwater superoleophobicity for oil/water

separation. *J. Membr. Sci.* **566**, 268-277 (2018).

17. Li, R. *et al.* Inkjet printing assisted fabrication of polyphenol-based coating membranes for oil/water separation. *Chemosphere* **250**, 126236 (2020).

18. Sun, Y. *et al.* Surface hydrophilic modification of PVDF membranes based on tannin and zwitterionic substance towards effective oil-in-water emulsion separation. *Sep. Pur. Tech.* **234**, 116015 (2020).

19. Xie, A. *et al.* Photo-Fenton self-cleaning membranes with robust flux recovery for an efficient oil/water emulsion separation. *J. Mater. Chem. A* **7**, 8491-8502 (2019).

20. Cui, J. *et al.* Bio-inspired fabrication of superhydrophilic nanocomposite membrane based on surface modification of SiO_2 anchored by polydopamine towards effective oil-water emulsions separation. *Sep. Pur. Tech.* **209**, 434-442 (2019).

21. Wang, Z. *et al.* One-step transformation of highly hydrophobic membranes into superhydrophilic and underwater superoleophobic ones for high-efficiency separation of oil-in-water emulsions. *J. Mater. Chem. A* **6**, 3391-3396 (2018).

22. Yang, X. *et al.* Mussel-/diatom-inspired silicified membrane for high-efficiency water remediation. *J. Membr. Sci.* **597**, 117753 (2020).

23. Wang, Z. *et al.* Investigating and significantly improving the stability of tannic acid (TA)-aminopropyltriethoxysilane (APTES) coating for enhanced oil-water separation. *J. Membr. Sci.* **593**, 117383 (2020).

24. Zhao, Y. *et al.* Ultra-robust superwetting hierarchical membranes constructed by coordination complex networks for oily water treatment. *J. Membr. Sci.* **627**, 119234 (2021).

25. Yang, X. *et al.* Mussel-inspired structure evolution customizing membrane interface hydrophilization. *J. Membr. Sci.* **612**, 118471 (2020).

26. Lu, J. *et al.* Photocatalytically active superhydrophilic/superoleophobic coating. *ACS Omega* **5**, 11448-11454 (2020).

27. Wang, Z. *et al.* Separation of caustic nano-emulsions and macromolecular conformations with nanofibrous membranes of marine chitin. *ACS Appl. Mater. Inter.* **11**, 8576-8583 (2019).

28. Zhou, L. *et al.* Multifunctional filtration membrane with anti-viscous-oils-fouling capacity and selective dyes adsorption ability for complex wastewater remediation. *J. Hazard. Mater.* **413**, 125379 (2021).
